# Evaluation of naked-eye sensing and anion binding studies in *meso*-fluorescein substituted one-walled calix[4]pyrrole (C4P)†

**DOI:** 10.1039/d3ra08362d

**Published:** 2024-03-05

**Authors:** Shafieq Ahmad Wagay, Ufana Riaz, Manawwer Alam, Rashid Ali

**Affiliations:** a Department of Chemistry, Organic and Supramolecular Functional Materials Research Laboratory, Jamia Millia Islamia Okhla New Delhi 110025 India rali1@jmi.ac.in +91-7011867613; b Department of Chemistry and Biochemistry, North Carolina Central University 27707 USA; c Department of Chemistry, College of Science, King Saud University P. O. Box 2455 Riyadh 11451 Saudi Arabia

## Abstract

In this paper, we have design, synthesized and fully characterized a new *meso*-fluorescein substituted one-walled calix[4]pyrrole (C4P7), obtained from simple and easily available starting materials such as fluorescein, 4-hydroxyacetophenone and pyrrole. The anion sensing studies reveal that the C4P7 system displays selective and sensitive naked-eye sensing towards fluoride, phosphate, and acetate anions with the limit of detection of 4.27 mg L^−1^, 6.4 mg L^−1^, and 5.94 mg L^−1^, respectively. Moreover, the C4P7 receptor displays good results of binding (host–guest, 1 : 1) towards a variety of anions. The 1 : 1 binding stoichiometry was further confirmed by means of Job's plots. TD-DFT calculations showed that the HOMO–LUMO gap decreases in all the complexes (C4P7@anions) in comparison to the free C4P7 system. The authors are of the opinion that this work may provide a good platform to explore calix[4]pyrrole chemistry in the arena of recognition/sensing of biologically significant analytes in future studies.

## Abstract 2

Synthesis, anion binding studies and sensing properties of novel *meso*-fluorescien based one-walled calix[4]pyrrole has been reported.

## Graphical abstract

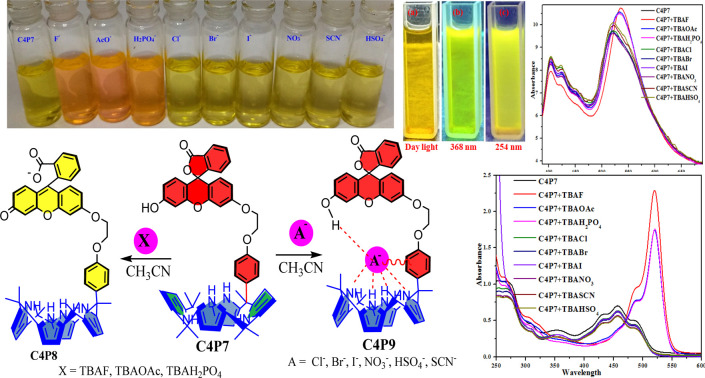

## Introduction

Supramolecular chemistry, introduces various artificial receptors which have revolutionized host–guest complexes to an advanced level in the past few decades. In the realm of supramolecular chemistry, sensors in general are devices that can help to detect and enumerate the physical and/or chemical aspects of the world around us in real time.^1–7^ Anions are considered as vital components of various biological systems, since they regulate and/or are responsible for a myriad of environmental and biological processes in our everyday life.^8–22^ Importantly, anionic species exist in almost 70% of all the active sites of enzymes – playing a key role in genetic information storage, controlling osmotic pressure, generation of electrical signals, maintaining cell volume and activating signal transduction pathways.^23–38^

As a result, anionic species in the environment might either be detrimental pollutants or indispensable for further growth. Therefore, from the last few decades, extensive research in the development of new luminous and/or colorimetric anionic sensors are being continuously accelerating – monitoring the function, concentration, and location of the anionic entities.^39–44^ To this context, Xu *et al.*, have reported a naphthalimide-calix[4]arene based effective fluoride sensor – displaying selective fluorescence ‘quenching effect’ only with the fluoride anion in acetonitrile, among the various examined tetrabutylammonium anion salts.^45^ From the Job's plot, they have observed a 1 : 1 stoichiometry binding of the receptor with fluoride ion. Moreover, the authors have also noticed a deprotonation of the naphthalimide NH, as the fluoride ion is sufficiently basic to deprotonate the NH-proton, causing a long wavelength color change. Markedly, ^1^H-NMR spectra also confirmed the deprotonation of NH-proton of the naphthalimide moiety.

On the other hand, Fabbrizzi and teammates have reported a versatile urea-based symmetrical bis-naphthalimide-appended selective fluoride sensor, which undergo stepwise deprotonation in DMSO – resulting a new intense band development *ca.* at 540 nm with decreases in the intensity of the original band at 400 nm, the color turning from yellow-to-red (red-shift).^46^ Noticeably, on further addition of [Bu_4_N]F, the color turned into the blue from red, and the band at 540 nm disappeared whileas another new broad band was formed at around 600 nm. In any case, the reversible successive deprotonation of NH protons was confirmed, as directed by the fact that, on gradual addition of water, the blue solution turned first into the red and then to yellow color. Moreover, such successive deprotonation was also confirmed by virtue of ^1^H-NMR by these workers.

In a separate study, Fu's research group has revealed a vital guanidiniocarbonyl pyrrole functionalized 4-amino-1,8-naphthalimide fluorescent chemosensory material, which showed selective fluorescent enhancement with pyrophosphate in aqueous solution over the other anionic entities like ATP, AMP, and ADP.^47^ Liu *et al.*, have reported two macrocyclic bis-benzimidazolium salts based receptors for the sensitive and selective detection of acetate and nitrate anions.^48^ Quite recently, a small molecule, *N*-tertbutyldimethylsilyl-3,6-diiodocarbazoles a conjugated organic dye, was design, constructed and employed as a selective fluoride colorimetric sensor by Zheng's research group.^49^ The limit of detection (LOD) for this system was found to be 3 × 10^−5^ M. Notably, in the year of 2023, our research group has also reported a valuable fluorescein functionalized dipyrromethane (DPM) based chemosensor for the selective detection of fluoride, phosphate and acetate ions over various other tested anions.^50^

From the anion binding perspective, calix[4]pyrrole (C4P) and its congeners remained among the most studied macrocyclic systems for the selective recognization of neutral molecules, anions, and/or ion-pairs. This is because of their simple-synthetic procedures, besides they also have an appropriate cavity as well as conformational flexibility.^51–57^ Remarkably, since the Sessler's research group, investigated the anion binding study for the first time of the parent C4P system in 1996,^58^ this versatile receptor rekindled pervasive interest of the researchers worldwide, as it has several possible positions (*e.g. meso*-, β-pyrrolic positions, & NH sites) to functionalize with ease.^59–66^ Therefore, taking the advantage of these tunable positions, and also to further advance the C4P-chemistry to advance level. Recently, we have revealed anion binding studies of phthalimide-based one-walled C4P architecture through the UV-vis spectroscopy and time-dependent density functional theory (TD-DFT) calculations.^67^

On the other hand, in another work, our group in collaboration with P.-E. Danjou (France), has reported the chemodosimetric detection of the hydrazine molecule by utilizing the β-dicyanovinyl substituted C4P system.^68^

Although, in recent years, huge advancements and innovations have successfully been made in the field of chemosensors for selective and sensitive detection/identification of the physiologically significant anions.^69^ But, we still believe that there is always a pressing demand to design and construct novel simple-to-make yet effective sensory materials of specific interest. In the present paper, we have designed and synthesized a novel *meso*-substituted one-walled C4P7 receptor, and investigated its anion binding/sensing properties with the involvement of F^−^, Br^−^, Cl^−^, I^−^, AcO^−^, NO_3_^−^, HSO_4_^−^, SCN^−^, and H_2_PO_4_^−^ anions, used as their tetrabutylammonium (TBA) salts in acetonitrile solvent. Remarkably, results have revealed that C4P7 shows colorimetric sensing with F^−^, AcO^−^, and H_2_PO_4_^−^, attaining a light orange color from yellowish in MeCN. In present work, the C4P7 moiety acts as the binding part whileas the fluorescein portion – acting as a signaling unit. Remarkably, the designed supramolecular structure acts as an anion sensor (*e.g.*, for F^−^, AcO^−^, and H_2_PO_4_^−^) as well as anionic receptor for various other tested anions. Here, the C4P moiety is responsible for interacting and binding the anions, whereas the fluorescein moiety undergoes a detectable change in response to the binding/sensing of anions. Moreover, the receptor C4P7 displayed a 1 : 1 binding behavior (calculated by the means of an online supramolecular Bindfit v0.5 program) with all the tested anions, which were further confirmed from the Job's method, also known as a method of continuous variation.

## Results and discussion

### Synthesis and characterization

**Scheme 1:**
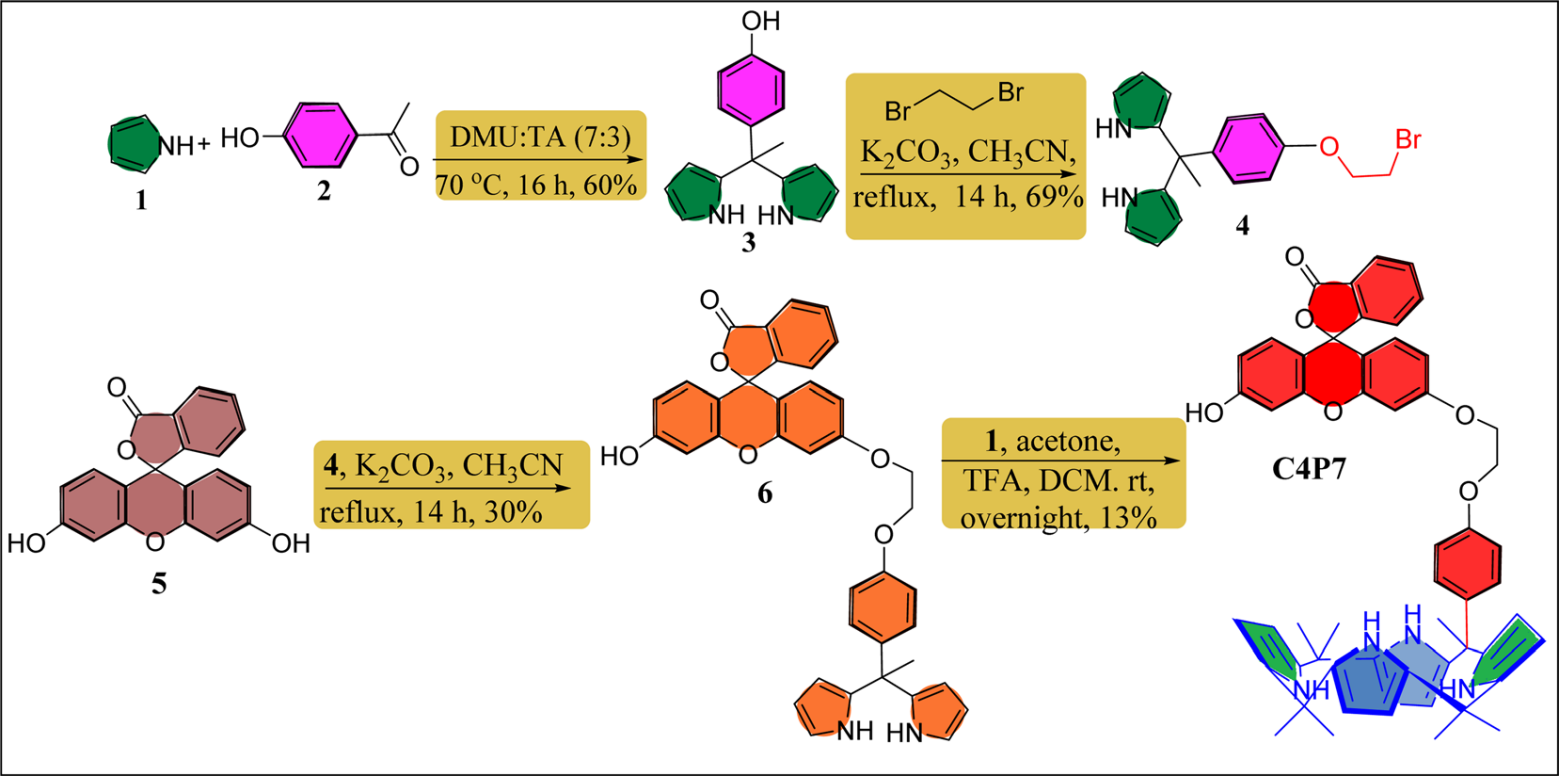
Synthetic route for the construction of *meso*-fluorescein substituted one-walled C4P7.

Fluorescein functionalized one-walled calix[4]pyrrole (C4P7) has been prepared through multistep reaction from easily available starting materials like fluorescein, 4-hydroxyacetophenone, acetone and freshly distilled pyrrole. To summarize, we began with the synthesis of dipyrromethane (DPM) 3 by condensing 4-hydroxyacetophenone with a freshly distilled pyrrole under eco-friendly condition using a 7 : 3 ratio of *N*,*N*′-dimethylurea (DMU) and l-(+)-tartaric acid (TA) at 70 °C using our earlier reported procedure.^52^ Next, the DPM 4 was achieved in good yield (69%) from the reaction of DPM 3 with 1,2-dibromoethane in the presence of K_2_CO_3_/MeCN under refluxing condition for 14 h. Later, thus prepared DPM 4 was coupled with parent fluorescein 5 using K_2_CO_3_ as a base to deliver the fluorescein functionalized DPM 6 in 30% yield. Lastly, an acid-catalyzed macrocyclization of the functionalized DPM 6 with freshly distilled pyrrole and acetone was accomplished to furnish the desired *meso*-fluorescein functionalized C4P7 in 13% yield (Scheme 1). The confirmation of the structure of C4P7 as well as the intermediate compounds were done by means of the standard spectroscopic techniques (see ESI†).

### Naked-eye visualization of the color change (sensing studies)

**Fig. 1:**
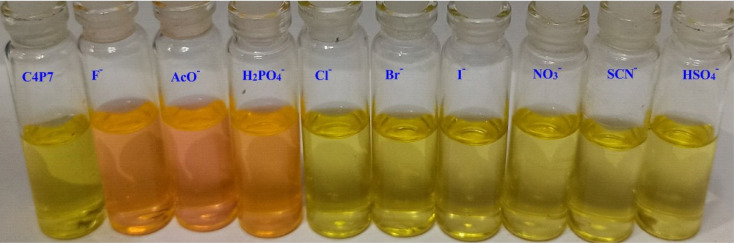
Naked-eye color change visualization of the C4P7 receptor upon the addition of TBA salts of a variety of anions in CH_3_CN.

The colorimetric anion sensing of fluorescein functionalized C4P7 was determined through the naked-eye, UV-vis and fluorescence experiments in acetonitrile solution. Initially, the colorimetric experiments were performed in which 60 μL of TBA salts (4.0 × 10^−3^ M) of F^−^, Cl^−^, Br^−^, I^−^, SCN^−^, NO_3_^−^, AcO^−^, HSO_4_^−^, and H_2_PO_4_^−^ anions, were separately added into the 2.5 mL of C4P7 (0.5 × 10^−5^ M) in acetonitrile solution. Interestingly, a clear change in the color from light yellow to orange was observed by the naked-eye with AcO^−^, F^−^, and H_2_PO_4_^−^, whereas no color change was seen upon the addition of other examined anions under identical conditions, even by increasing the addition of anionic solution up to 100 μL (Fig. 1).

**Fig. 2:**
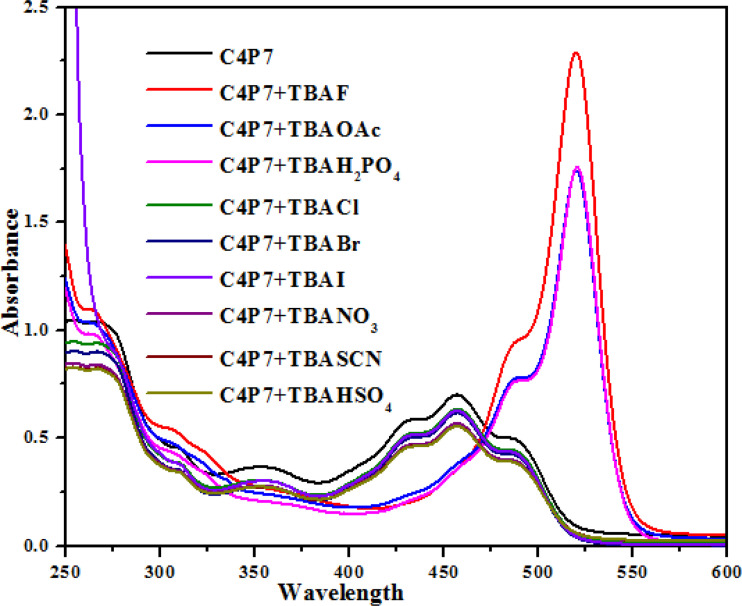
UV-vis spectra of C4P7 (0.5 × 10^−5^ M) after addition of 100 μL TBA salts (4.0 × 10^−3^ M) of various anions in CH_3_CN solution.

To further support these experiments, the UV-vis studies of C4P7 system (0.5 × 10^−5^ M) with 100 μL of tetrabutylammonium salts (4.0 × 10^−3^ M) of the anticipated anions were accomplished distinctly. Inspection of the Fig. 2, clearly illustrate that the free C4P7 receptor with Cl^−^, Br^−^, I^−^, SCN^−^, NO_3_^−^, and HSO_4_^−^ anions exhibit a triplet like strong absorption band with high band intensity *ca.* at 458 nm, and two weak intensity shoulders at 489 nm and 431 nm, besides a low intensity band at 356 nm. Pleasingly, a large red-shift was perceived in the UV-vis spectrum upon addition of F^−^, AcO^−^, and H_2_PO_4_^−^ anions – developing a new intense band at 524 nm at the expense of the original triplet like band having the maxima at 458 nm (Fig. 2).

**Fig. 3:**
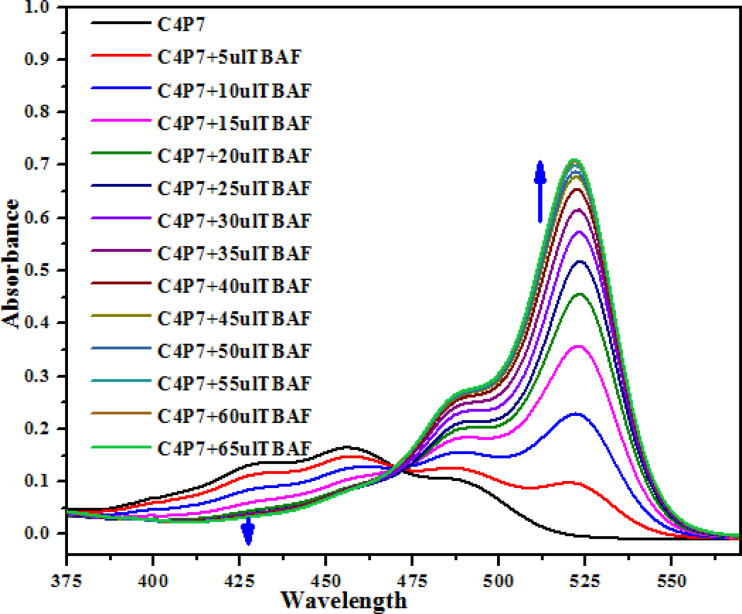
The UV-vis spectra of C4P7 (0.5 × 10^−5^ M) with TBAF (4.0 × 10^−3^ M) in CH_3_CN.

**Scheme 2:**
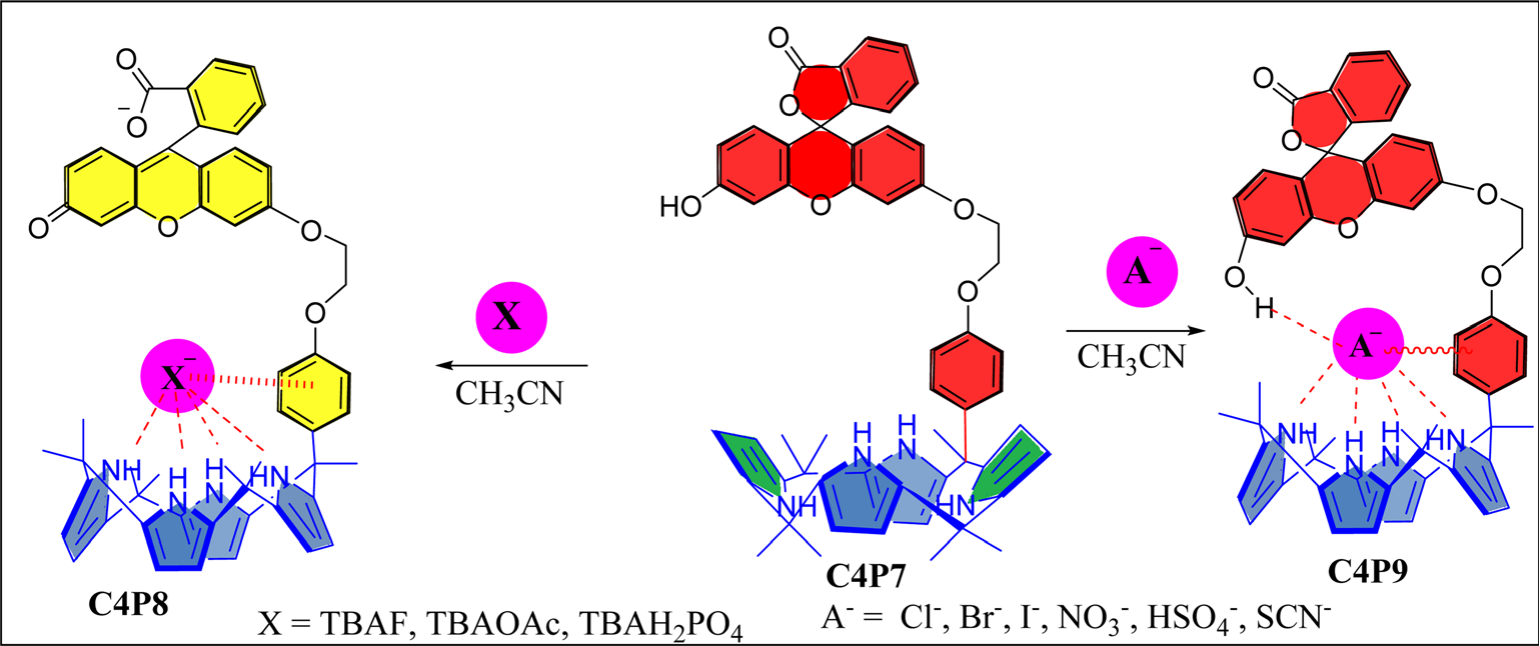
Plausible schematic representation of the C4P7 with anionic salts.

Next, UV-vis titration studies in CH_3_CN solvent of the fluorescein functionalized C4P7 with several anions were investigated in order to elucidate the binding/sensing behavior. Titrations were made successful with continuous addition of the solution of anions such as F^−^, Cl^−^, Br^−^, I^−^, SCN^−^, NO_3_^−^, AcO^−^, HSO_4_^−^ and H_2_PO_4_^−^ (used as their tetrabutylammonium salts). The appearance of a new intense band *ca.* at 524 nm, represent a bathochromic shift using the anions F^−^ anion in comparison to the free receptor C4P7 (Fig. 3). It can clearly be seen from the Fig. 3 that gradual addition of TBAF solution, showed a continuous increase in the absorption intensity (hyperchromic shift) at 524 nm, whereas simultaneous decrease in the intensity at 430 nm and 453 nm with the development of an isosbestic point *ca.* at ∼471 nm. The isosbestic point, clearly indicate that the C4P7 receptor with F^−^/AcO^−^/H_2_PO_4_^−^ is present in the equilibrium state. Interestingly, similar results were also obtained with C4P7@CH_3_COO^−^ as well as C4P7@H_2_PO_4_^−^ under identical conditions (see ESI†). On contrary, no such observations were detected with other test anions like Cl^−^, Br^−^, I^−^, SCN^−^, NO_3_^−^, and HSO_4_^−^ (see ESI†). This unique behavior of F^−^, AcO^−^, and H_2_PO_4_^−^ may be due to their more basic nature as compared to the other examined anions, resulting in the abstraction of a phenolic proton, thereby leading towards the ring-opening of the spirolactone present in the fluorescein moiety to produce a coloured quinonoid structure C4P8 (Scheme 2).^70,71^ To further confirm the binding and/or sensing behavior of this C4P7 system, we have also performed the comparative ^1^H-NMR experiments (Fig. S7, see ESI†), which reveal the abstraction of the phenolic proton – confirmed through the disappearance of OH peak, and binding of the F^−^ ion with the calix framework through hydrogens bonds (Fig. S7,† downfield shift in the NH-protons) as depicted in the Scheme 2.

**Fig. 4:**
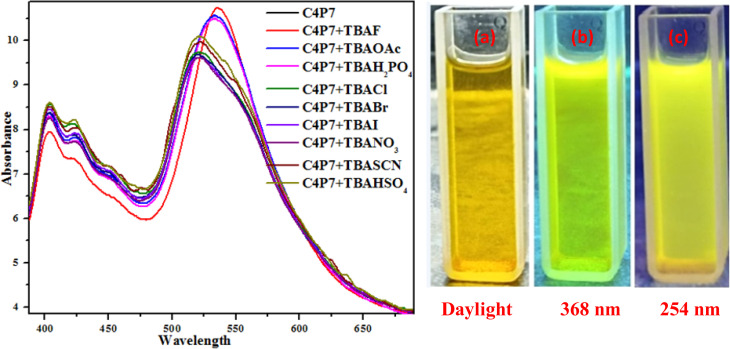
Fluorescence spectra of C4P7 (0.25 × 10^−5^ M) after the addition of 100 μL TBA salt (2.0 × 10^−3^ M) of various anions (left panel), and the solution phase images of C4P7 (right panel) (a) daylight; (b) UV-light (368 nm); (c) UV-light (254 nm).

**Fig. 5:**
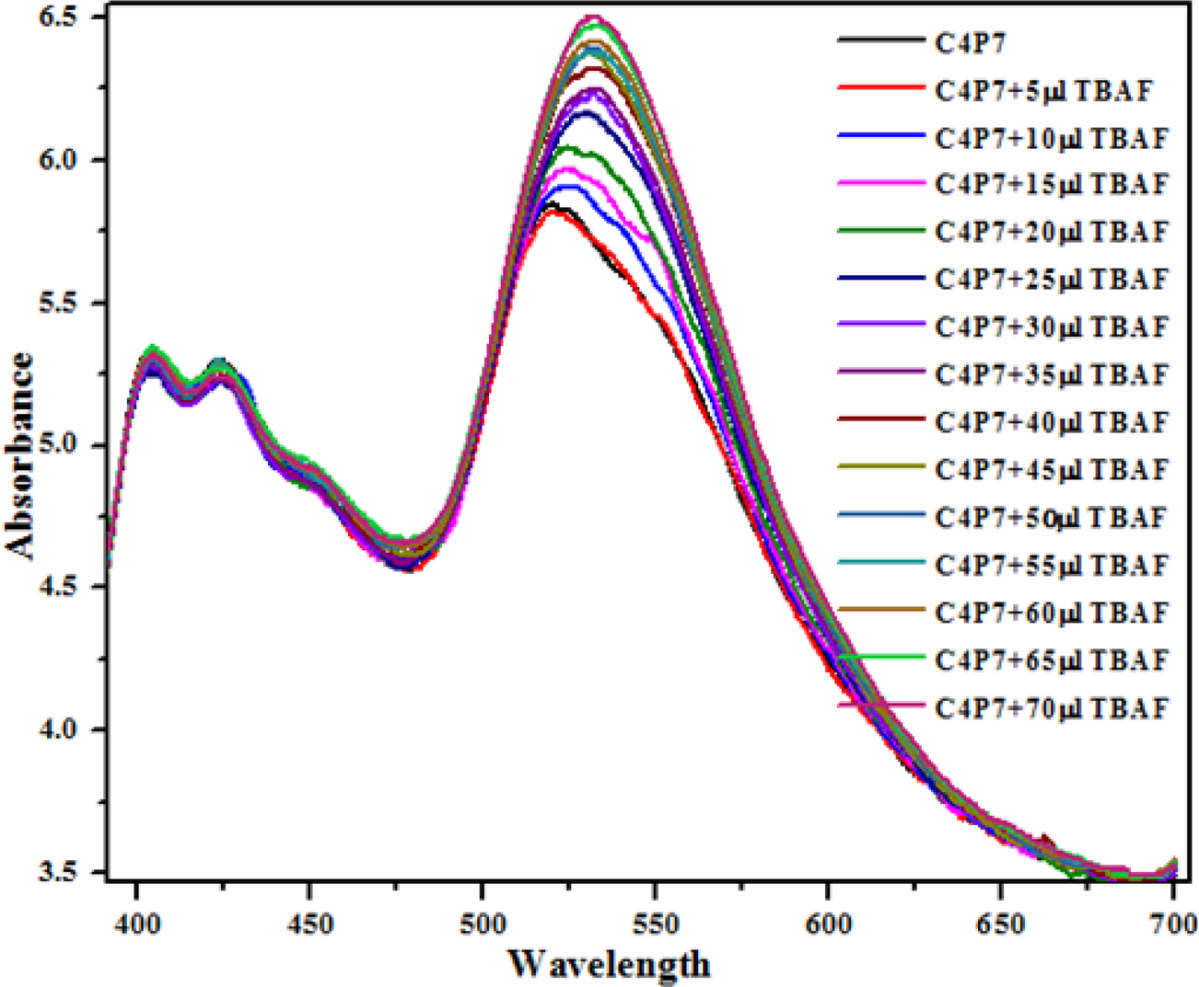
Fluorescence titration emission spectra of the C4P7 (0.25 × 10^−5^ M) with TBAF (2.0 × 10^−3^ M) in CH_3_CN.

Moreover, fluorescence studies of the receptor C4P7 (0.25 × 10^−5^ M) with the aforesaid anions (2 × 10^−4^ M), used as their tetrabutylammonium (TBA) salts in acetonitrile were also accomplished to further support the naked-eye as well as UV-vis experiments (Fig. 4). To further elucidate the sensing results, fluorescence titrations of the C4P7 with 0–100 μL solution of F^−^, CH_3_COO^−^, and H_2_PO_4_^−^ were performed in acetonitrile solvent (Fig. 5 and see ESI†). It can be seen from the Fig. 5 that the free receptor C4P7 shows emission bands at 402, nm 423 nm, and 520 nm. From the comparison of fluorescence analysis, it could clearly be seen that the spectra displayed a shift in wavelength from (520 nm to 536 nm), (520 nm to 532 nm), and (520 nm to 531) nm in the presence of 100 μL of F^−^, CH_3_COO^−^, and H_2_PO_4_^−^ anions, respectively, whereas other anions were unable to show any spectral change in the fluorescence spectra (Fig. 4 and see ESI†). Importantly, our results were in accordance with the earlier similar types of outcomes.^55,72–74^ Moreover, solution phase images reveal that the C4P7 display a light green and yellowish fluorescence upon illuminating with the UV-lamp at 368 nm and 254 nm, respectively (Fig. 4a–c).

### Limit of detection (LOD) and limit of quantification (LOQ)

The LOD/LOQ of the anticipated sensor C4P7 were calculated using the UV-vis titrations spectral data utilizing the eqn (1) and (2), respectively. For this, the first calibration curve was achieved from the plot of absorption intensity increment (*A* − *A*_0_) as a function of fluoride/phosphate/acetate anions concentrations (Table 1). The regression curve equation was afterward extended for the low concentration measure.^75^

1 LOD = 3.3 × S.D./*k*

2 LOQ = 10 × S.D./*k*

where S.D. is the standard deviation and *k* is the slope.

**Table 1:** Estimated values of the LOD and LOQ for TBAF, TBAH_2_PO_4_ and TBAOAc

Property	TBAF	TBAH_2_PO_4_	TBAOAc
LOD	4.27 mg L^−1^	6.4 mg L^−1^	5.94 mg L^−1^
LOQ	12.95 mg L^−1^	19.41 mg L^−1^	18.01 mg L^−1^

### Evaluation of anion binding studies

**Fig. 6:**
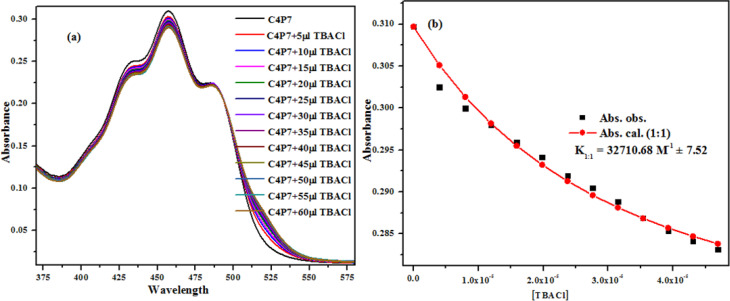
UV-vis titration (a) of the receptor C4P7 with TBACl in CH_3_CN, and binding isotherm (b) fitting of UV-vis titration data using Bindfit v0.5 program.

To investigate the anion binding studies of C4P7 receptor (0.5 × 10^−5^ M), the UV-vis titrations were achieved with various anions used as their tetrabutylammonium salts (4.0 × 10^−3^ M) in acetonitrile solution. Initially, the spectrum of free receptor C4P7 (0.5 × 10^−5^ M) was recorded after that solution of the guest TBACl (4.0 × 10^−3^ M) was added in fractions (5 μL) into it, and the spectra of each titrant was documented until the saturation point was reached, showing a notable change in the complexed (C4P7@Cl) form with a *λ*_max_ at 457 nm (Fig. 6a). The data was fitted *ca.* at *λ*_max_ 457 nm through the online supramolecular Bindfit v0.5 program, revealing the formation of a 1 : 1 host–guest complex with a binding constant *K*_1:1_ = 32 710.68 M^−1^ (Fig. 6b). For other anions as well, the same procedure was applied to evaluate the stoichiometry and binding constants (see ESI†), and it can clearly be seen the formation of 1 : 1 complexes with all the tested anions, showing moderate to good binding affinities (Table 2). Noticeably, comparison of the binding strength of the present studies with the earlier reported ones, showed better results, may be due to some secondary non-covalent supramolecular interactions.^41,52,67^

**Table 2:** The binding constant values of C4P7 with various anions

S. no.	Anion	C4P7 (*K*_a_ M−1)
1	Cl−	32 710.68 ± (7.52)
2	Br−	14 068.96 ± (6.42)
3	I−	68.63 ± (4.52)
4	NO_3_−	7055.87 ± (4.03)
5	SCN−	5114.57 ± (2.02)
6	HSO_4_−	1106.59 ± (1.99)

**Fig. 7:**
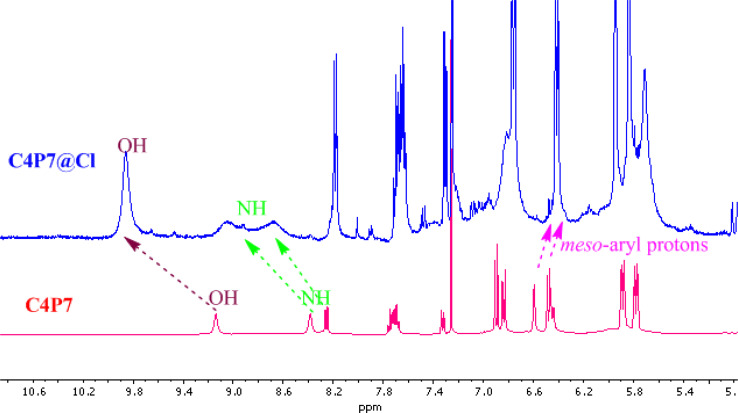
Comparative ^1^H-NMR spectra of C4P7 & complex (C4P7@Cl^−^) recorded in CDCl_3_.

As shown in Fig. 7, the OH proton signal appeared as a broad singlet at *δ* 9.14 ppm shifted downfield to *δ* 9.94 ppm, displaying the O–H–anion interaction. On the other, the NH protons also downfield shifted from *δ* 8.49 ppm to *δ* 9.12 ppm and *δ* 8.25 ppm to *δ* 8.78 ppm, which clearly indicate that the anion is bound through hydrogen bonding. However, the *meso*-substituted aromatic protons display slight upfield shift from *δ* 6.59 ppm to *δ* 6.44 ppm and *δ* 6.48 ppm to *δ* 6.41 ppm, which confirms anion–π interaction.^50,53^ Besides, these observation, β-pyrrolic protons of the C4P7 also showed upfield shift – revealing the change in the conformation of C4P7 from 1,3-alternate to the cone conformation.

**Fig. 8:**
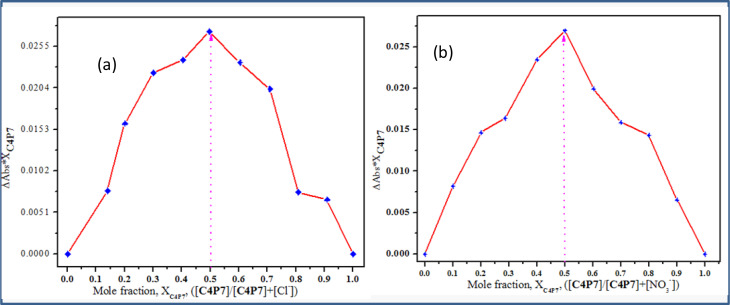
Job's plots of C4P7 with (a) TBACl and (b) TBANO_3_ in acetonitrile at temperature 30 °C ± 2 °C. These plots were plotted between the change in absorbance and the mole-fraction of C4P7*vs.* the mole fraction of TABCl/TBANO_3_.

To further validate the stoichiometric results obtained from the online supramolecular Bindfit v0.5 program for C4P7 with various anions, the method of continuous variation (Job's method) was also used, plotting the graphs against the change in absorption *vs.* the mole-fraction of the guest molecules (Fig. 8). The concentration of both the guests (anionic entities) and the host molecule (C4P7) were held constant in acetonitrile solution at temperature 30 °C ± 2 °C, and their mole fraction were varied. It can be inspected from the Fig. 8 that the concentrations of C4P7@Cl^−^ as well as C4P7@NO_3_^−^ approaches to a maximum value at 0.5, confirming the formation of 1 : 1 complexes between C4P7 and the tested anions. Therefore, results from the online supramolecular Bindfit v0.5 program and Job's plots are in complete agreement, depicting the formation of 1 : 1 complexes of the newly constructed C4P7 system with the assessed anions.

### Computational studies

On the other hand, to further support the experimental results, computational findings of C4P7 with different anions were executed utilizing the Gaussian 09 software, and the geometry optimizations for ground state (*S*_o_) were accomplished by means of Becke's exchange functional coalesced with the “Lee–Yang–Parr correlation functional” designated as B3LYP. Whileas, the UV-vis absorption studies were computed through TD-B3LYP technique taking into the consideration of solvent effects through “conductor polarizable continuum model” (CPCM). Vibrational frequencies were measured to elucidate the attained geometries correspond to the minima onto the potential energy surface deprived of any imaginary frequencies. For all the optimized geometries, the time dependent density functional theory (TD-DFT) calculations were presented at the same level to develop the fundamental ‘excited states’ (ESs) of the C4P7. Moreover, vertical ionization energies have also been estimated at the identical level of theory. The building complex were adjusted at B3LYP/6-31G(d) in both gas phase as well as in solution phase and with “basis set superimposition error” (BSSE). Counterpoise correction equation of the Boys–Bernardi was employed to revenue into the description of the BSSE.^67,76^ This mode was involved to measure the binding energies of the anionic complexes contemplating complex compound as a fragment A and anionic species as the fragment B through following equation:DeBSSE = (AB) = *E*(AB)AB − *E*(AAB) −*E*(BAB)Herein, *E*(ABAB) = ‘energy of the complex AB with the basis set of AB’; whileas *E*(AAB) and *E*(B+) are the energies of fragments A and B, respectively, with the basis set of AB at their relevant geometries embraced from the complex of AB.

**Fig. 9:**
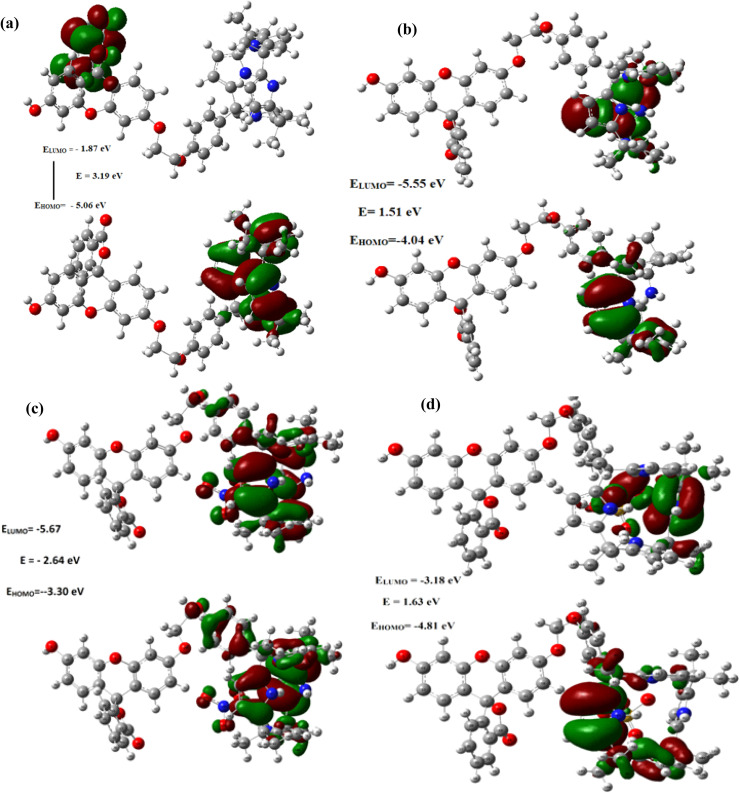
Frontier molecular orbitals distribution of (a) C4P7 (b) C4P7@TBACl (c) C4P7@TBANO_3_ and (d) C4P7@TBAHSO_4_ calculated using the TD-DFT with 6-31G (d) basis set.

**Fig. 10:**
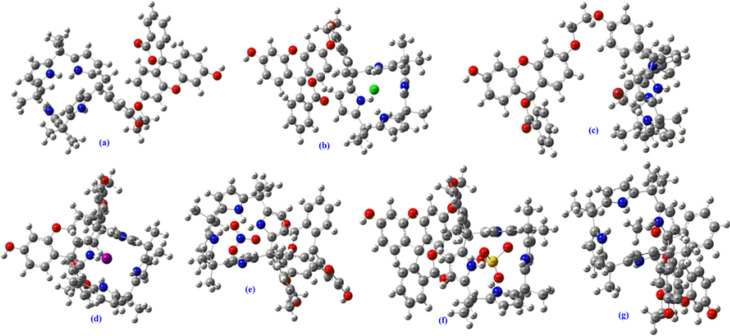
DFT optimized structures of C4P7 (a), and C4P7@anions (b–g) using 6-31G(d) basis set. The counter cation TBA has been omitted for clarity.

Remarkably, as can be seen from the Fig. 9 and S43–48,† the HOMO–LUMO gap were found be decreased after the complexations of C4P7 with different anions (C4P7@anions), and the energy gap were found to be in the order of: C4P7 (3.19 eV) > C4P7@SCN^−^ (2.90 eV) > C4P7@NO_3_^−^ (2.64 eV) > C4P7@HSO_4_^−^ (1.63 eV) > C4P7@Cl^−^ (1.51 eV) > C4P7@Br^−^ (1.05 eV) > C4P7@I^−^ (1.02 eV). Moreover, it is to be pointed out that in the case of parent C4P7 (Fig. S44†) as well as C4P7@I (Fig. S46†), the HOMO lies on the calix framework, whileas the LUMO exist onto the fluorescein subunit. On the other hand, in most of the complexes (C4P7@anions), both HOMO and LUMO exits onto the calix frame except C4P7@SCN (Fig. S48†) in which both HOMO and LUMO exists onto the fluorescein unit (Fig. S48†). The optimized structures using B3LYP/6-31G(d) for the parent C4P7 and its complexes with different anions are depicted in the Fig. 10 and S36–42.†

## Conclusions

In this particular contribution, we have successfully synthesized a novel *meso*-fluorescein functionalized one-walled calix[4]pyrrole (C4P7) system, which was confirmed by means of the standard spectroscopic techniques (*e.g.*, ^1^H-NMR, ^13^C-NMR & HRMS). Receptor C4P7 demonstrated the selective colorimetric sensing towards fluoride, phosphate and acetate anions with the limit of detections (LODs) 4.27 mg L^−1^, 6.4 mg L^−1^, and 5.94 mg L^−1^, and limit of quantifications (LOQs) 12.95 mg L^−1^, 19.41 mg L^−1^, and 18.01 mg L^−1^, respectively. The naked-eye color change was observed from dark yellow to orange in acetonitrile solution for the above three test anions, whileas no such observation was noticed with several other examined anions.

The selective sensing with F^−^, AcO^−^, and H_2_PO_4_^−^, may be due to strong basic nature of these anions as compared to the other assessed anions, leading to the deprotonation of the phenolic OH-group. And subsequent ring-opening of the spirolactone to produce the coloured quinonoid structure C4P8. Moreover, the receptor C4P7 have also showed worthy binding results (1 : 1 complex formation) with all the inspected anions in acetonitrile solution. Finally, the 1 : 1 bonding stoichiometry was further validated through the Job's plots, and the involvement of OH/NH were confirmed by means of partial ^1^H-NMR spectra.

The HOMO–LUMO gap were found be reduced after the complexations of C4P7 with tested anions (C4P7@anions), and the energy gap were found in the order: C4P7 (3.19 eV) > C4P7@SCN^−^ (2.90 eV) > C4P7@NO_3_^−^ (2.64 eV) > C4P7@HSO_4_^−^ (1.63 eV) > C4P7@Cl^−^ (1.51 eV) > C4P7@Br^−^ (1.05 eV) > C4P7@I^−^ (1.02 eV). Moreover, in the case of parent C4P7 and C4P7@I, the HOMO lies on the calix framework whilesas the LUMO exist onto the fluorescein moiety. On the other hand, in all other cases, both HOMO and LUMO exists onto the calix network except C4P7@SCN, in which both HOMO and LUMO exists onto the fluorescein unit. We believe that the current results may be valid for the recognition and/or sensing of several biologically important molecules in future studies.

## Conflicts of interest

There are no conflicts to declare.

## Supplementary Material

RA-014-D3RA08362D-s001
