# Supplementary material for: Evaluation of naked-eye sensing and anion binding studies in meso-fluorescein substituted one-walled calix[4]pyrrole (C4P)
Source: RSC Adv. 2024 Mar 5;14(11):7786–96. doi: 10.1039/d3ra08362d (PMC10912976; doi:10.1039/d3ra08362d)
Supplement: RA-014-D3RA08362D-s001 [file RA-014-D3RA08362D-s001.pdf]

Electronic Supplementary Material (ESI) for RSC Advances.
This journal is © The Royal Society of Chemistry 2024

Evaluation of naked-eye sensing and anion binding studies in *meso*-fluorescein substituted one-walled calix[4]pyrrole (C4P)

Shafieq Ahmad Wagay[a], Ufana Riaz[b], Manawwer Alam[c] & Rashid Ali[a]*

[a]Organic and Supramolecular Functional Materials Research Laboratory, Department of Chemistry, Jamia Millia Islamia, Okhla, New Delhi 110025, India;
Phone: +91-7011867613; Email: rali1@jmi.ac.in
[b]Department of Chemistry and Biochemistry, North Carolina Central University, 27707, USA
[c]Department of Chemistry, College of Science, King Saud University, P.O. Box 2455, Riyadh 11451, Saudi Arabia

Table of contents

(i) Characterization part (^{1}H-NMR, ^{13}C-NMR, HRMS of all the synthesized compounds),

(ii) Anion sensing.

(ii) Anion binding studies.

(iv) Frontier molecular orbital distribution of **C4P7** with various anions and their theoretically predicted FTIR spectrum.

Experimental method:

General information

The chemicals employed to synthesize fluorescein-functionalized calix[4]pyrrole were purchased from different chemical companies such as Merck, GLR, LOBA Chemie, CDH, SRL, and Spectrochem. Throughout the reaction, the pyrrole was freshly used after distilled at atmospheric pressure. The solvents were properly dried before their usage, and then utilized under an inert (N_2) atmosphere. Thin-layer chromatography (TLC), carried out on aluminium plates coated with silica gel in a variety of ethyl acetate and hexane mixtures for confirming the progress of reaction. The spots were developed under UV light or in an iodine chamber. Column chromatography was used to separate the synthesized compounds using silica gel with a mesh size of 100–200. With the use of $CDCl_3$/DMSO solvent, the isolated compounds were identified and verified using ^{1}H-NMR and ^{13}C-NMR (Bruker 400 MHz and Jeol 500 MHz spectrometers). The ^{1}H-NMR data of reported compounds were compared to the literature reported ones. In terms of tetramethylsilane (TMS), the chemical changes are represented in parts per million (ppm) units. Utilizing a Perkin Elmer UV-vis spectrophotometer, the UV-vis titration experiments were carried out in HPLC grade acetonitrile at 30 °C ± 2 °C with standard concentrations of host and guest molecules. The UV-vis spectra obtained between 700 nm to 200 nm in range. The data was calculated by utilizing Nelder-Mead fit method from online supramolecular Bindfit v0.5 program.[1,2] On the other hand, the fluorescence titration measurements were performed in HPLC grade acetonitrile within standard concentration of receptor **C4P7** and guest molecules at 30°C ±2°C by utilizing BK-F96pro fluorescence spectrophotometer instrument and the spectra were recorded between 700 and 400 nm. The compounds **(3)** and **(4)** are synthesized according to reported procedures.[3,4]

Synthesis of fluorescein functionalized dipyrromethane (6): Fluorescein **5** (3 mmol**)**, compound**4** (7.5 mmol), and K_2CO_3 (24 mmol) were mixed in acetonitrile (50 ml) and the resulting mixture was refluxed under nitrogen atmosphere for 12 hours. The completion of the reaction was monitored by TLC and allows the reaction mixture to cool down to room temperature, after that the acetonitrile was removed under reduced pressure. To the resulting brownish oily residue, ethyl acetate (50 ml) and water (100 ml) was added, and the organic layer was separated off and washed twice with 50 ml of water. The organic layer was then dried over anhydrous Na_2SO_4 and the solvent was evaporated in vacuum to give a dark red solid, which was purified by column chromatography over silica gel 25%

ethyl acetate hexane solution furnished the desired red solid compound **6** (30% yield).^{1}H-NMR (400 MHz, DMSO-d_6) δ 10.33 (s, 1H), 7.99 (d, *J* = 7.6 Hz, 2H), 7.75 (dd, *J* = 7.5Hz, 3H), 7.26 (s, 1H), 6.69 (d, *J* = 1.4 Hz, 3H), 6.58 (m, 2H), 6.55 (s, 3H), 5.86 (m, 2H), 5.72 (t, *J* = 1.7 Hz, 2H), 2.23 – 2.16 (m, 2H), 1.99 –1.94 (m, 2H), 1.50 (s, 6H).^{13}C-NMR (101 MHz, DMSO-d_6) δ 172.81, 165.82, 151.00, 150.63, 140.57, 138.25, 134.80, 131.66, 129.38, 127.85, 117.19, 110.44, 110.14, 106.87, 104.38, 49.07, 38.58, 36.24, 32.67, 25.26.HRMS (ESI) calculated for $C_{38}H_{30}N_2O_6H^+$: 611.2127, found: 611.2229.

Synthesis of fluorescein functionalized one walled calix[4]pyrrole (C4P7): To a stirred solution of **6**(115mg, 0.188mmol, 1equiv), acetone (0.11ml, 1.885 mmol, 10equiv), and pyrrole (0.065ml, 0.97mmol, 5 equiv.), in dry 30ml DCM was added TFA (0.043ml, 0.37mmmol, 3 equiv.) drop wise under an inert atmosphere in an ice cold temperature. After 30 minutes the reaction mixture was warmed to room temperature and stirred for 2 h. The completion of the reaction was monitored by TLC in 50% ethyl acetate and hexane, the reaction mixture was neutralized by 1 M NaOH and extracted with CH_2Cl_2.The organic phase was dried over Na_2SO_4, and concentrated under reduced pressure in vacuum to yield a crude red solid, which was later on subjected to column chromatography using by 1:1 ethyl acetate/hexane to give the desired orange colored fluorescein functionalized one walled C4P (13% yield) ^{1}H-NMR (400 MHz, $CDCl_3$) δ 9.14 (s, 1H), 8.47 (s, 2H),), 8.24 (s, 2H), 7.75 – 7.67 (m, 3H), 7.33 (dd, *J* = 7.3 Hz, 1H), 6.90 (d, J = 9.2 Hz, 2H), 6.84 (d, *J* = 6.9 Hz, 2H), 6.60 (d, *J* = 2.1 Hz, 2H), 6.48 (d, *J* = 8.9 Hz, 4H), 5.90 – 5.87 (m, 4H), 5.80–5.76 (m, 4H), 4.30 (s, 2H), 3.65 (s, 2H), 1.84 (s, 3H), 1.62 (s, 6H), 1.59 (s, 6H), 1.50 (s, 3H), 1.46 (s, 3H). ^{13}C-NMR (101 MHz, DMSO-d_6) δ 170.40, 165.44, 156.57, 150.92, 147.71, 141.56, 141.33, 138.49, 134.27, 133.67, 131.29, 131.18, 130.43, 130.06, 128.54, 117.54, 113.89, 106.68, 106.20, 65.69, 64.11, 56.96, 44.06, 28.66, 24.81, 22.42, 21.90; HRMS (ESI) calculated for $C_{55}H_{52}N_4O_6H^+$: 865.3960, found: 865.3421.

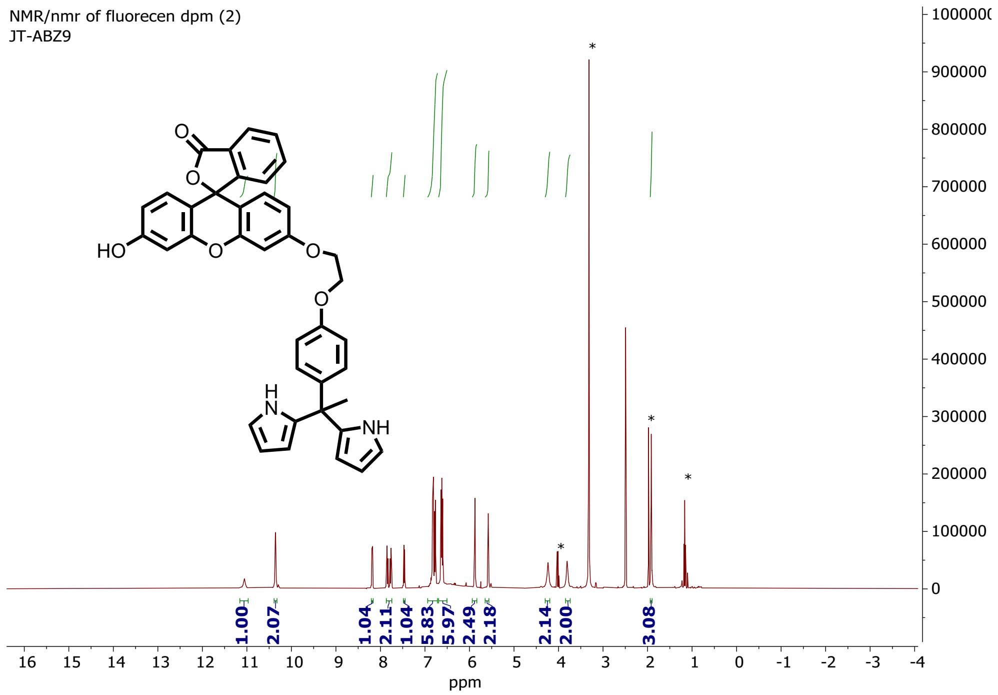

Figure S1. ^{1}H-NMR of fluorescein based-dipyrromethane derivative (**6**) recorded in DMSO-d^6.

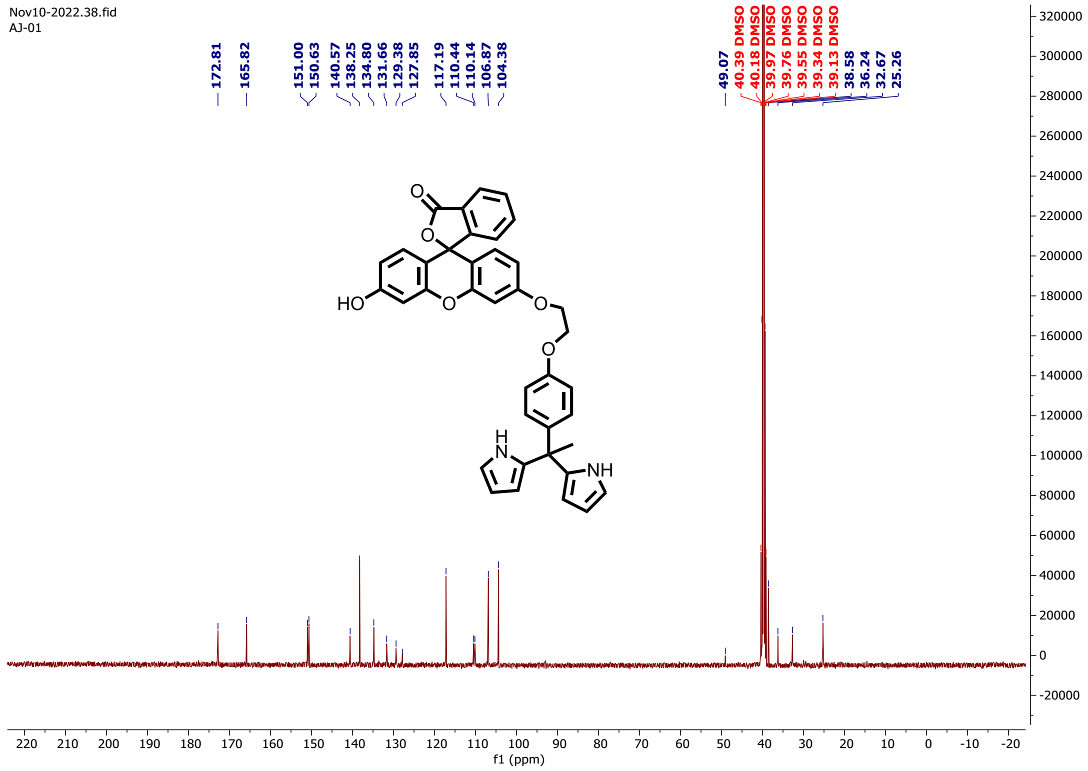

Figure S2. ^{13}C-NMR of fluorescein based dipyrromethane derivative (**6**) recorded in DMSO-d^6.

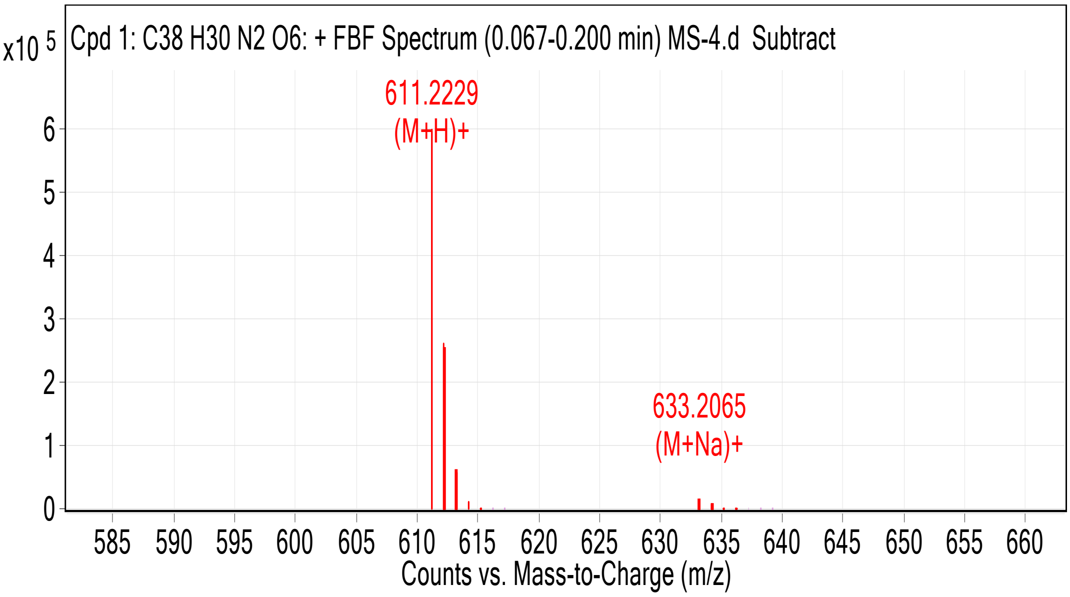

Figure S3. The HRMS of fluorescein based dipyrromethane derivative (**6**).

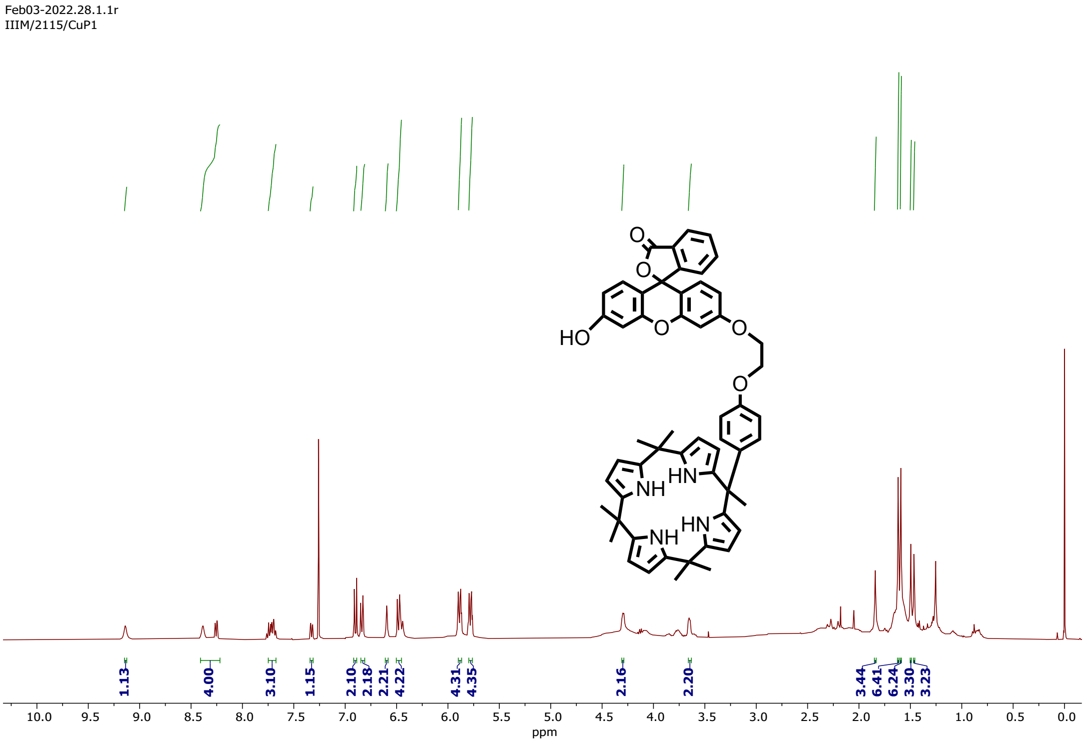

Figure S4. ^{1}H-NMR of fluorescein based one walled calix[4]pyrrole (**C4P7)** recorded in $CDCl_3$.

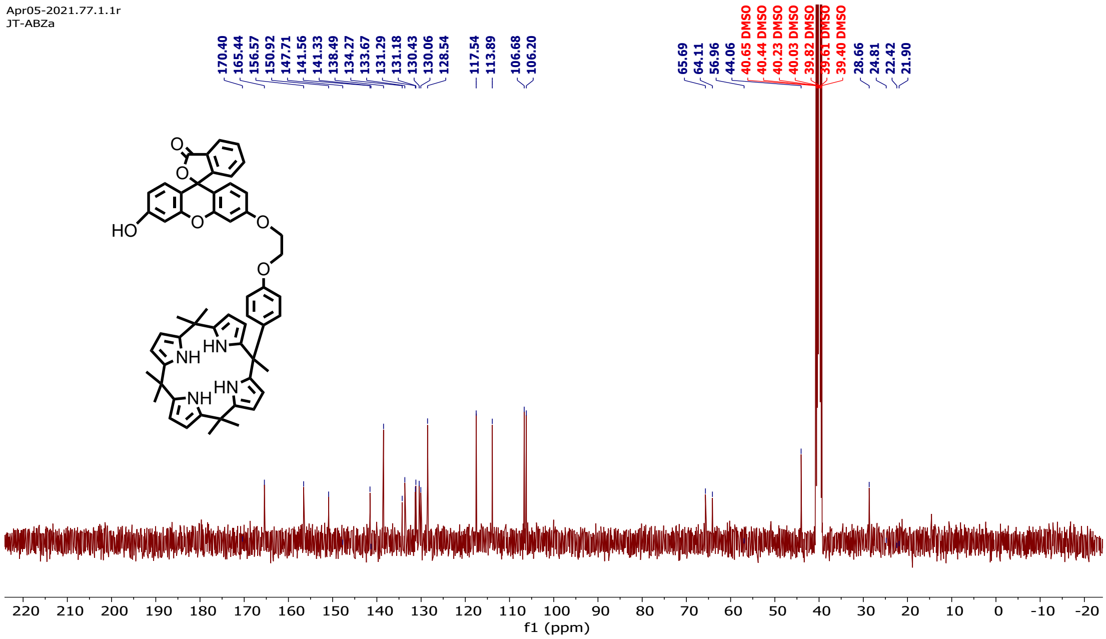

Figure S5. ^{13}C-NMR of fluorescein based one walled calix[4]pyrrole (**C4P7)** recorded in recorded in DMSO-d^6.

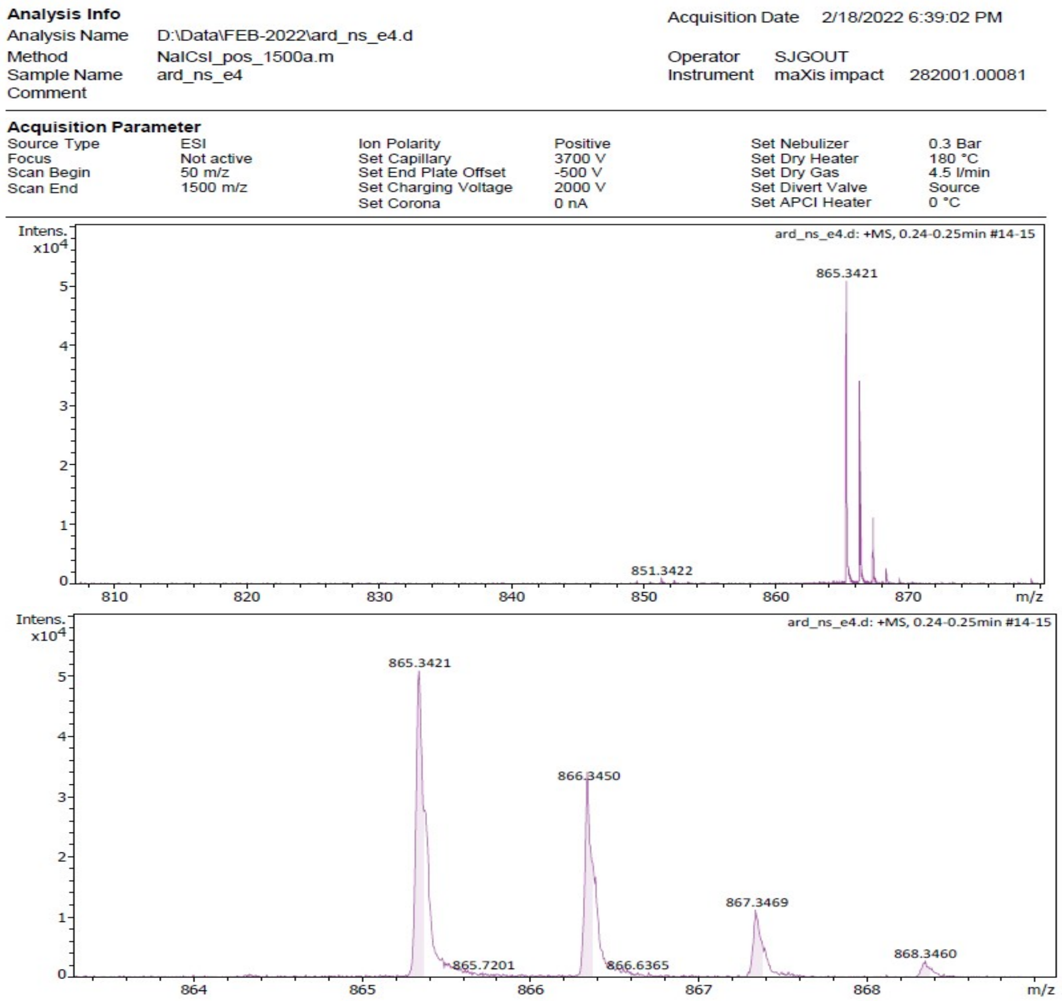

Figure S6. HRMS of fluorescein based one walled calix[4]pyrrole (**C4P7**).

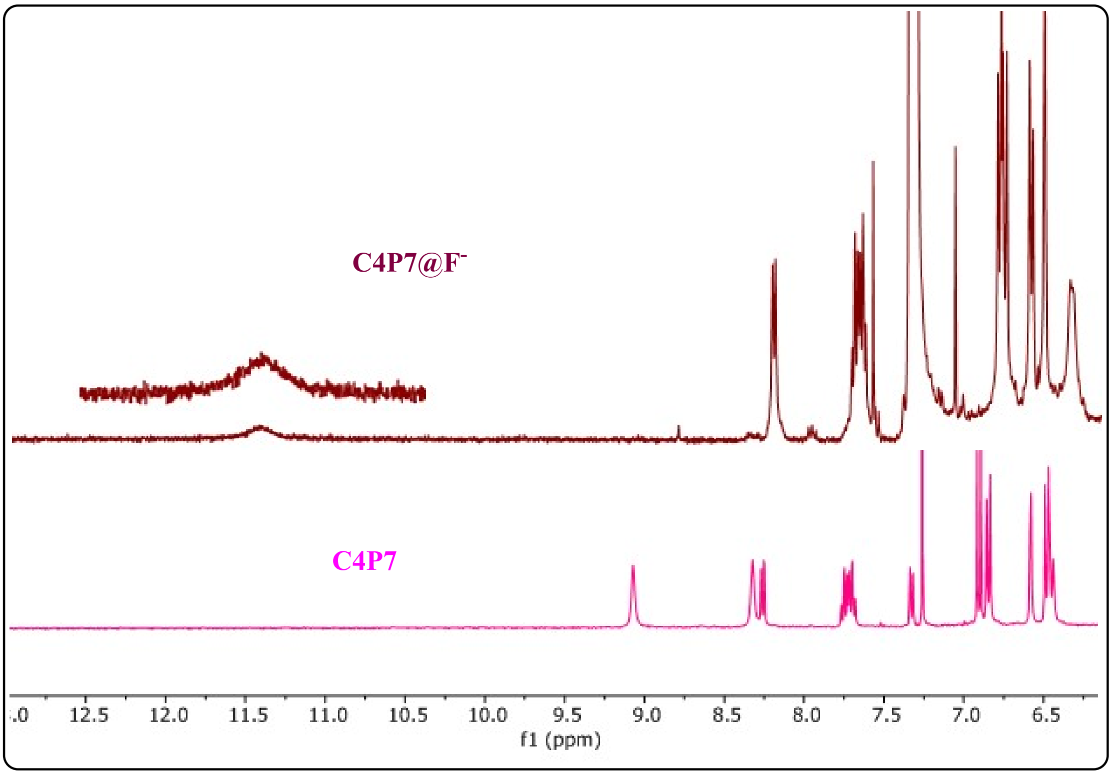

Figure S7. Comparative ^{1}H-NMR spectra of fluorescein based one-walled **C4P7** and its TBAF complex (i.e., **C4P7@F^{-}**) recorded in $CDCl_3$.

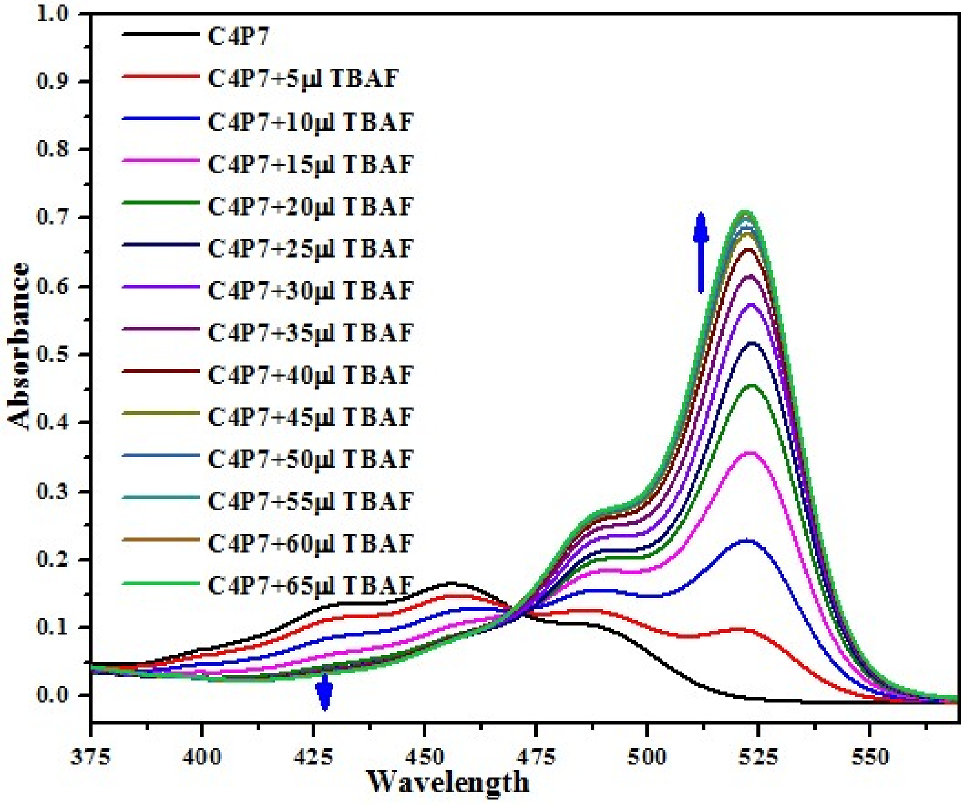

Figure S8. UV-vis titration absorption spectra of **C4P7** with TBAF in acetonitrile.

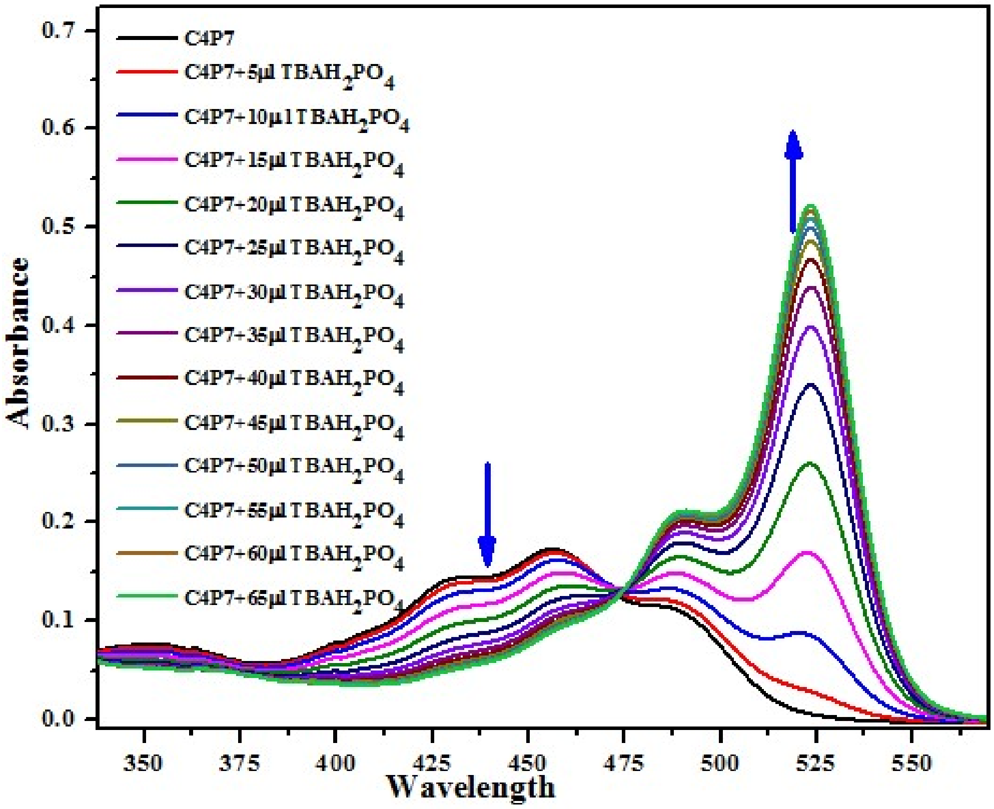

Figure S9. UV-vis titration absorption spectra of **C4P7** with $TBAH_2PO_4$ in acetonitrile.

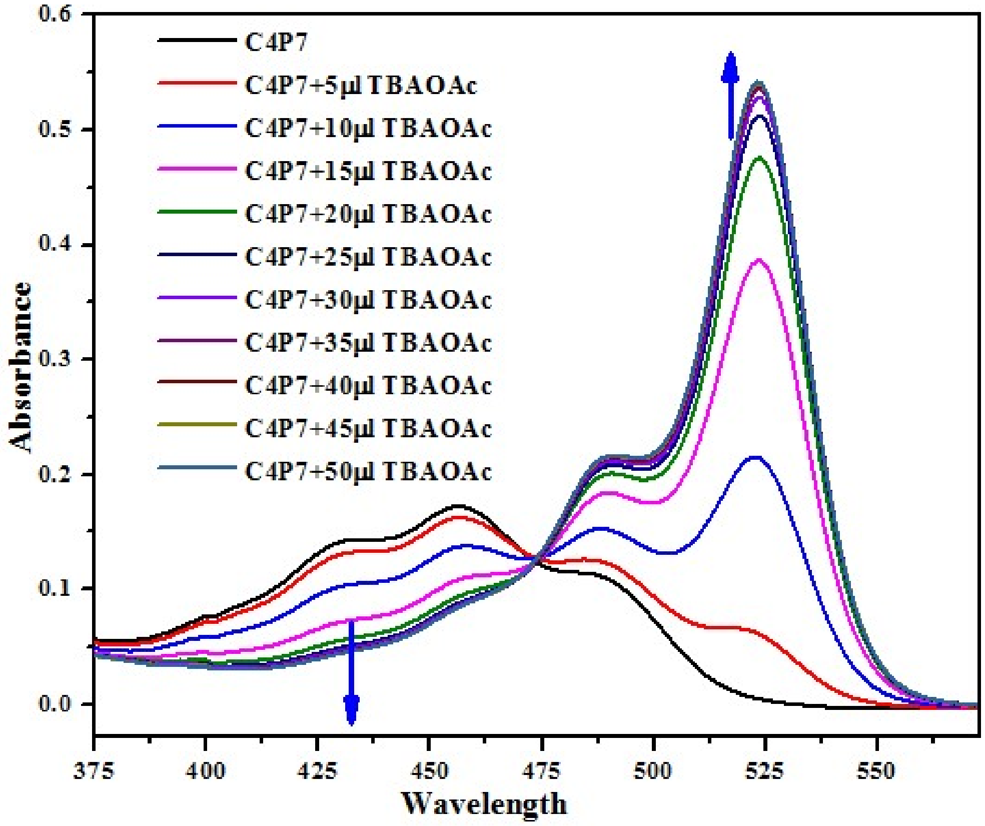

Figure S10. UV-vis titration absorption spectra of **C4P7** with TBAOAc in acetonitrile.

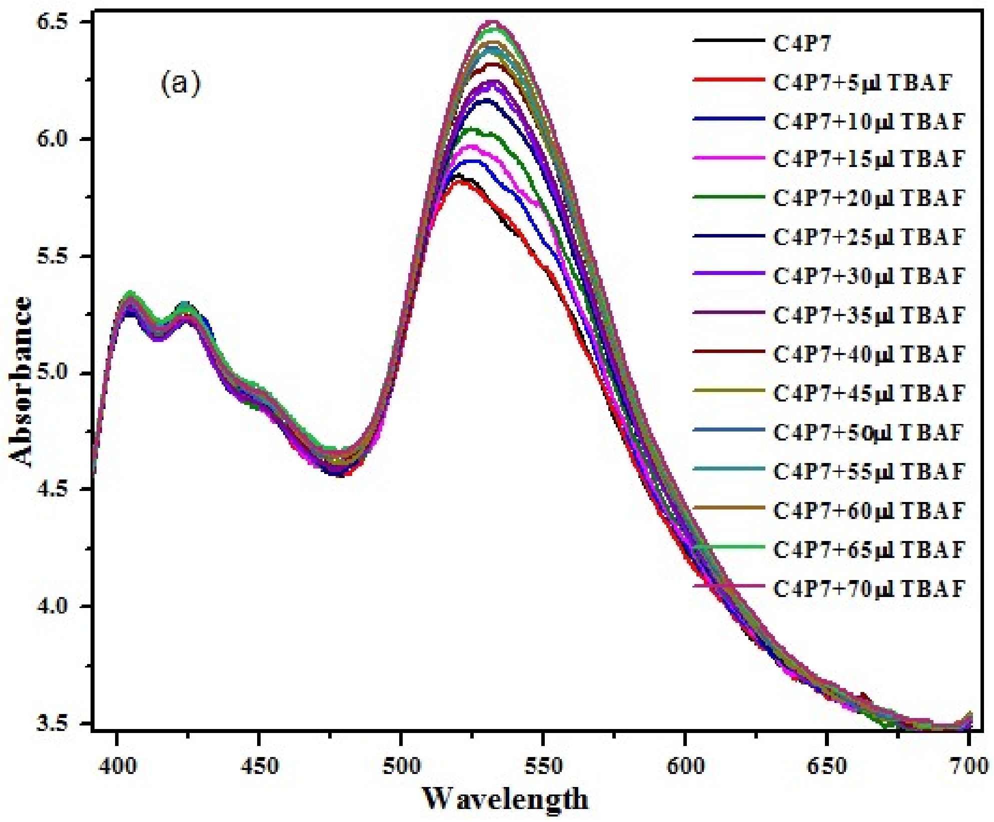

Figure S11. Fluorescence titration spectra of **C4P7** with TBAF in acetonitrile.

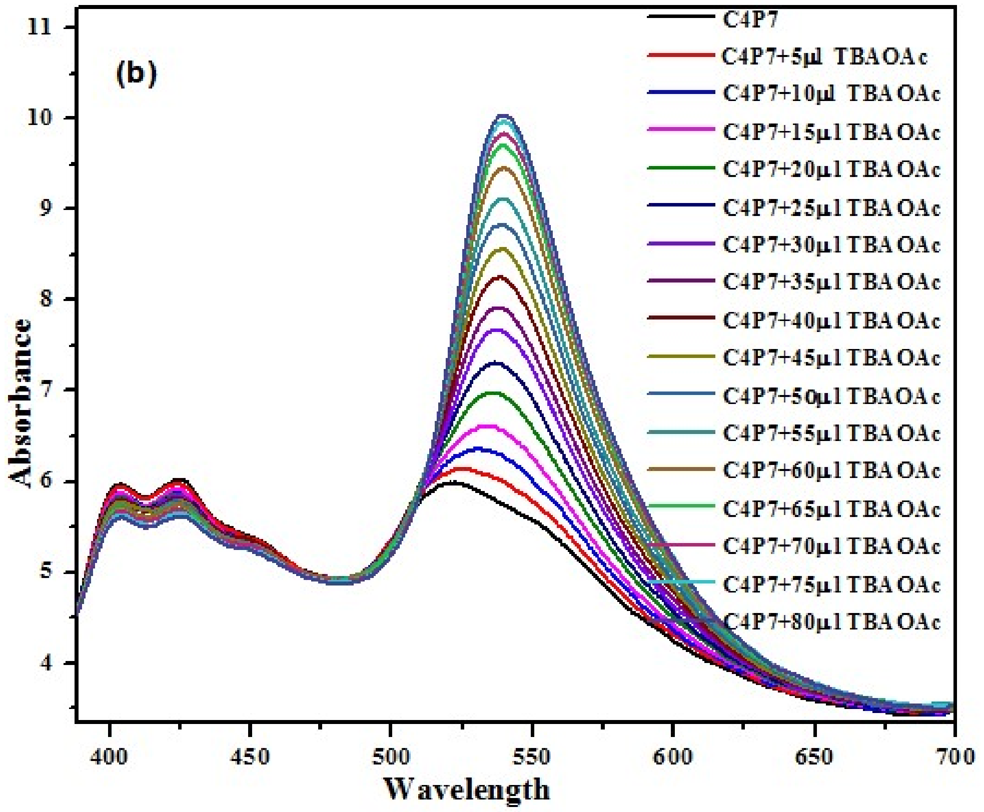

Figure S12. Fluorescence titration spectra of **C4P7** with TBAOAc in acetonitrile.

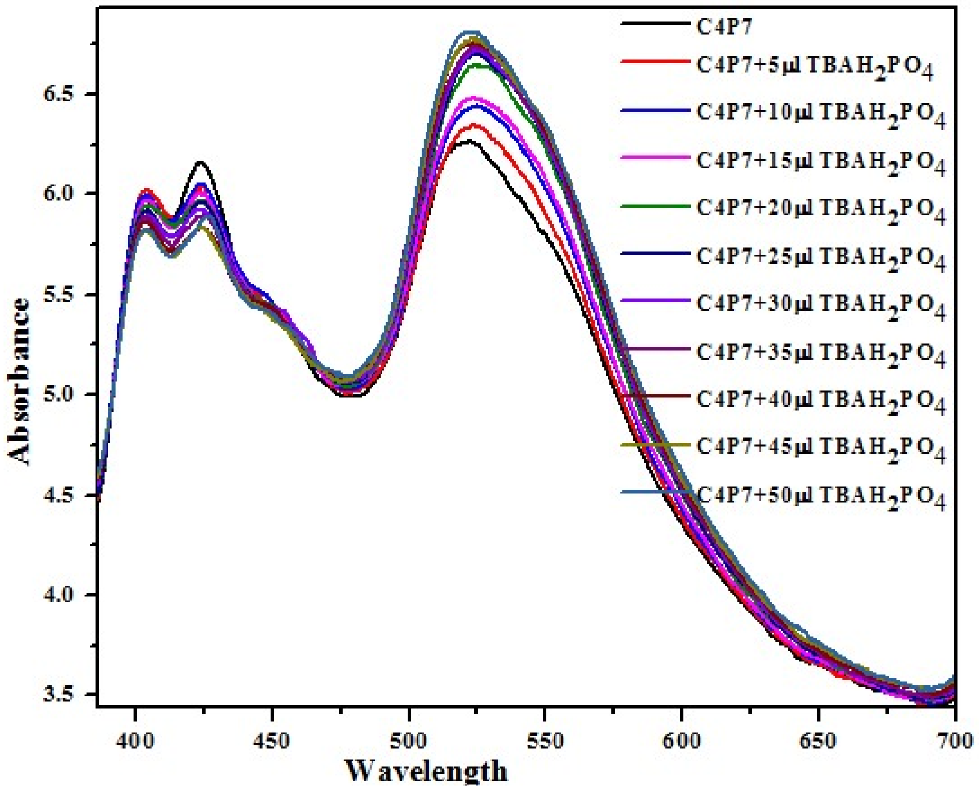

Figure S13. Fluorescence titration spectra of **C4P7** with $TBAH_2PO_4$ in acetonitrile.

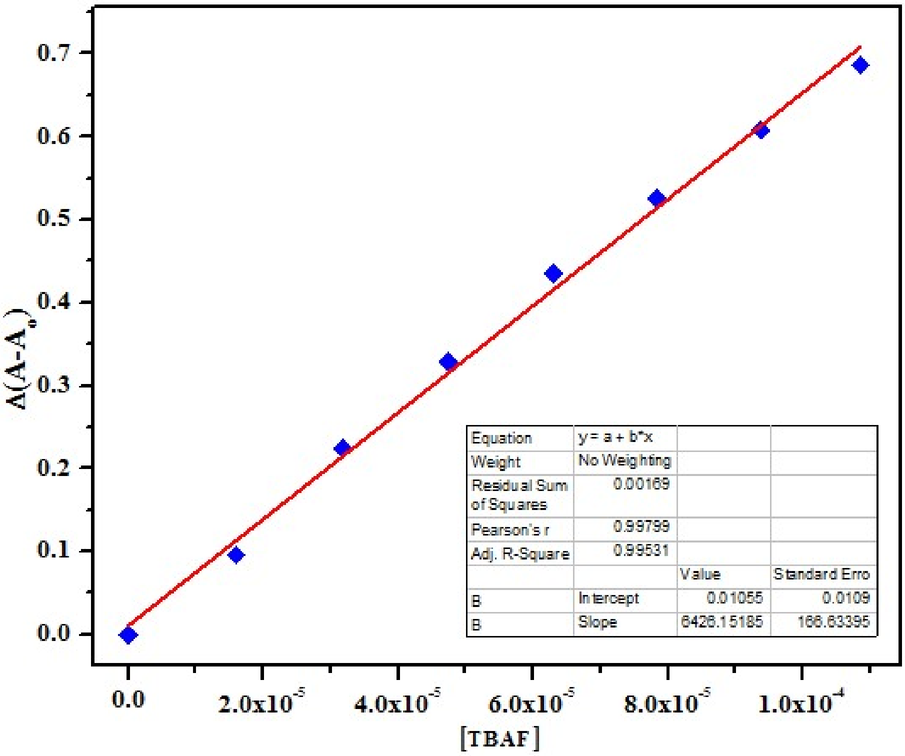

Figure S14. Calibration curves for monitoring the limit of detection and limit of quantification of F^{-} by **C4P7**.

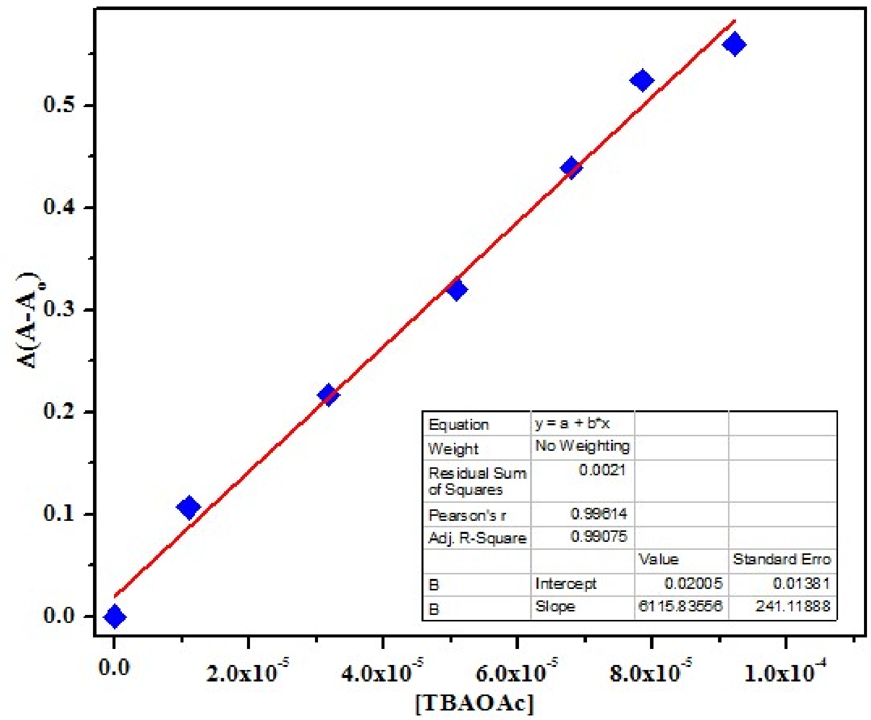

Figure S15. Calibration curves for monitoring the limit of detection and limit of quantification of $H_2PO_4^-$ by **C4P7**.

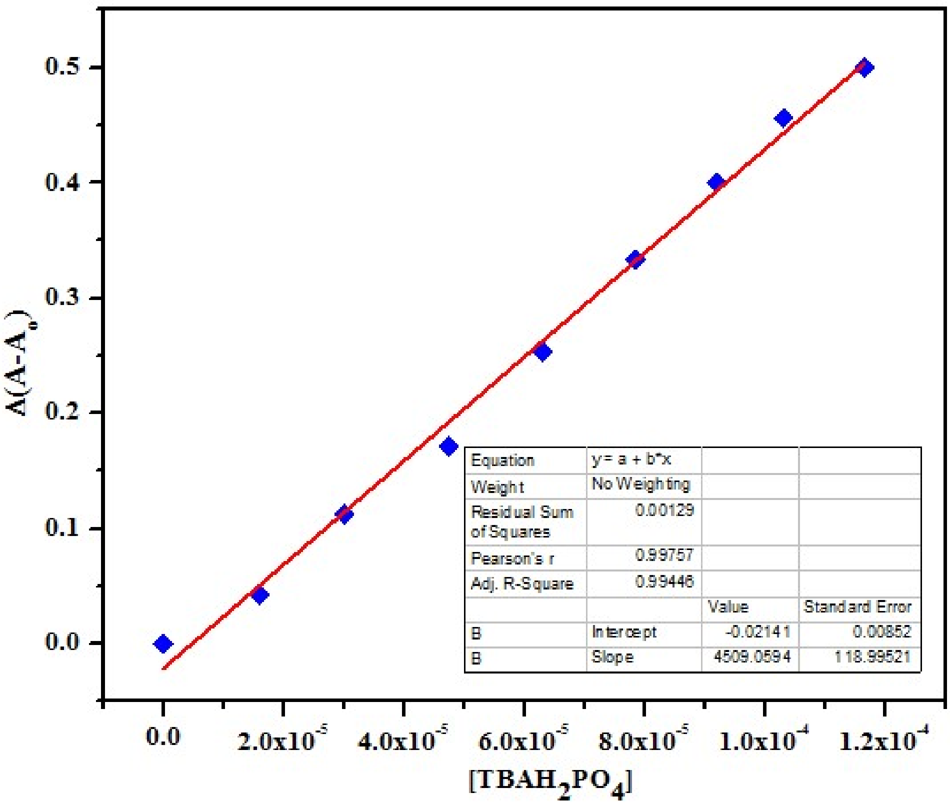

Figure S16. Calibration curves for monitoring the limit of detection and limit of quantification of $H_2PO_4^-$ by the receptor **C4P9**.

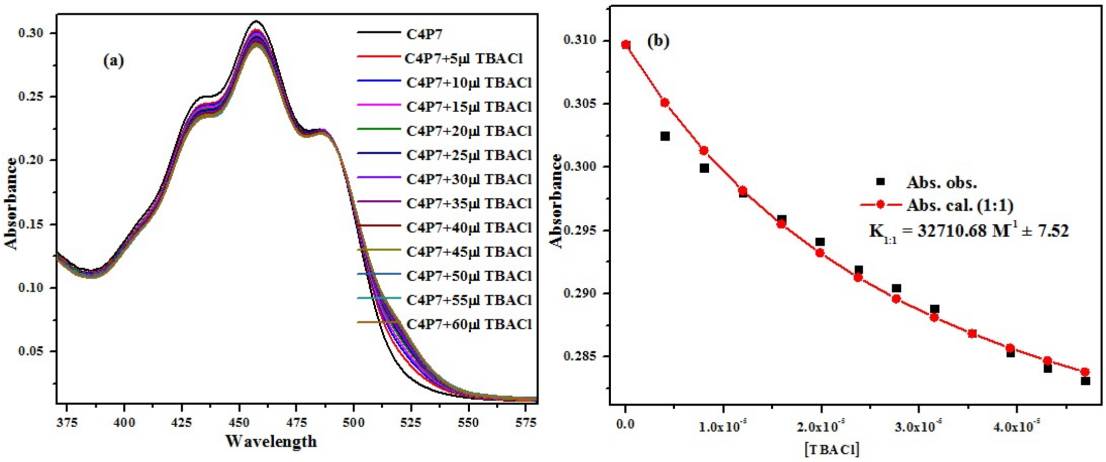

Figure S17. UV-vis titration of receptor **C4P7** with TBACl in CH_3CN and binding isotherm fitting of UV-vis titration data by using Bindfit v0.5 program.

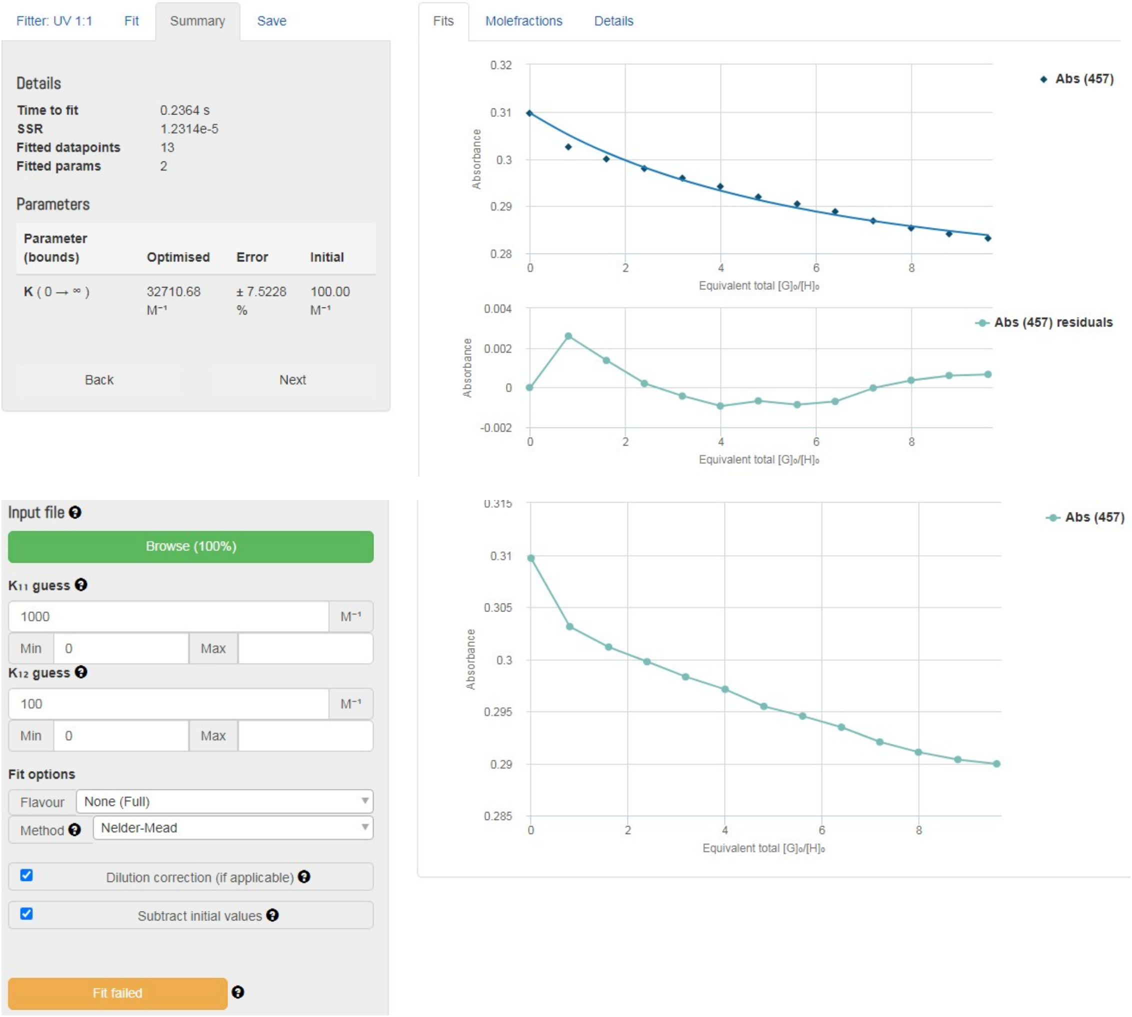

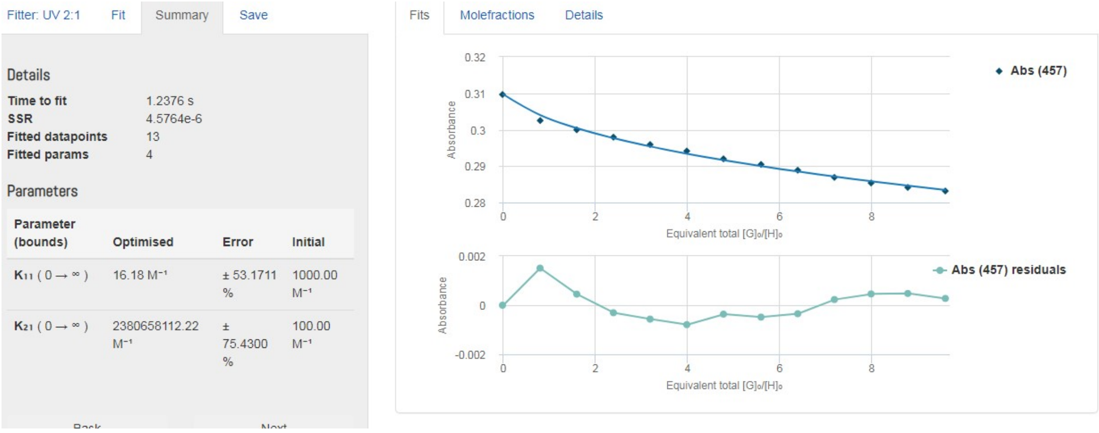

Figure S18. Snapshot capture of Bindfit plots for **C4P7**and TBACl titration, displaying 1:1 stoichiometry with satisfied value of K_{11}, error <7.52% utilizing Nelder-Mead fit.

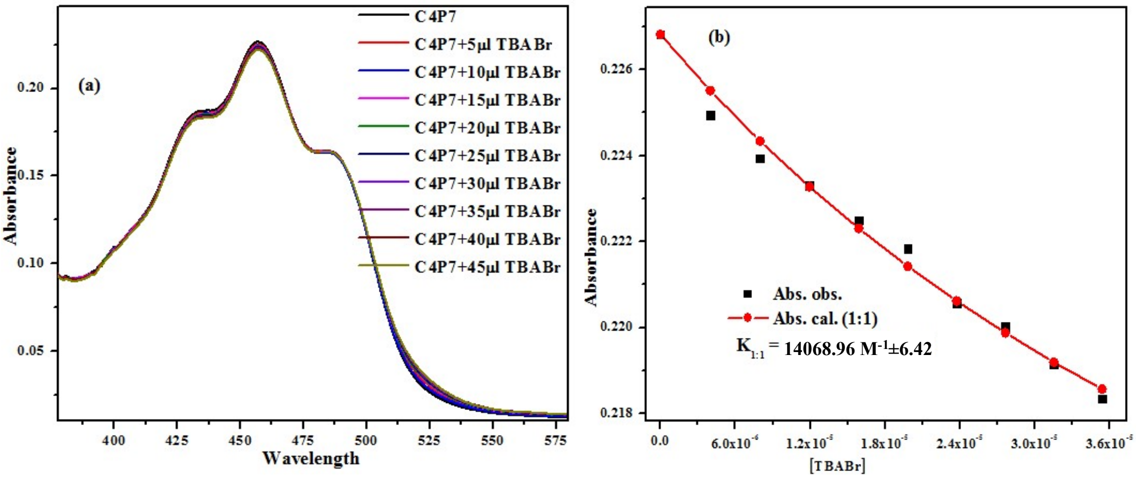

Figure S19. UV-vis titration of receptor **C4P7** with TBABr in CH_3CN and binding isotherm fitting of UV-vis titration data by using Bindfit v0.5 program.

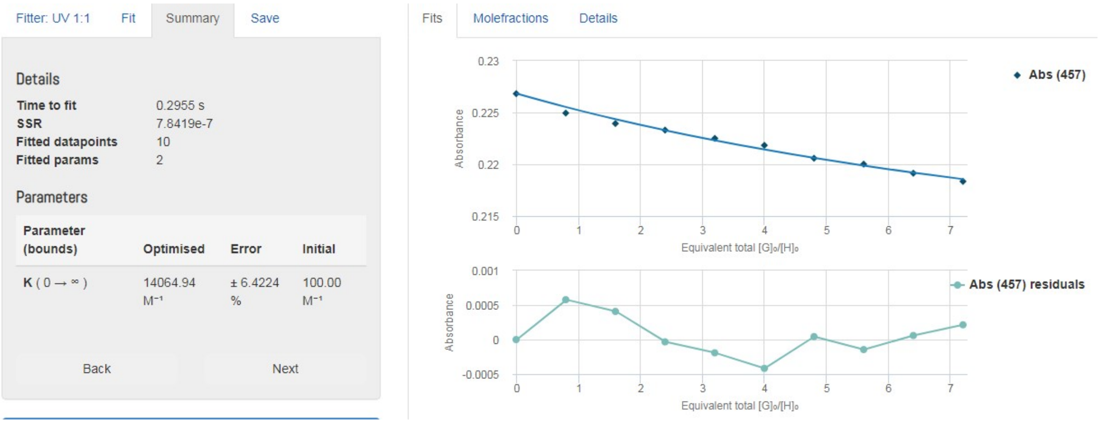

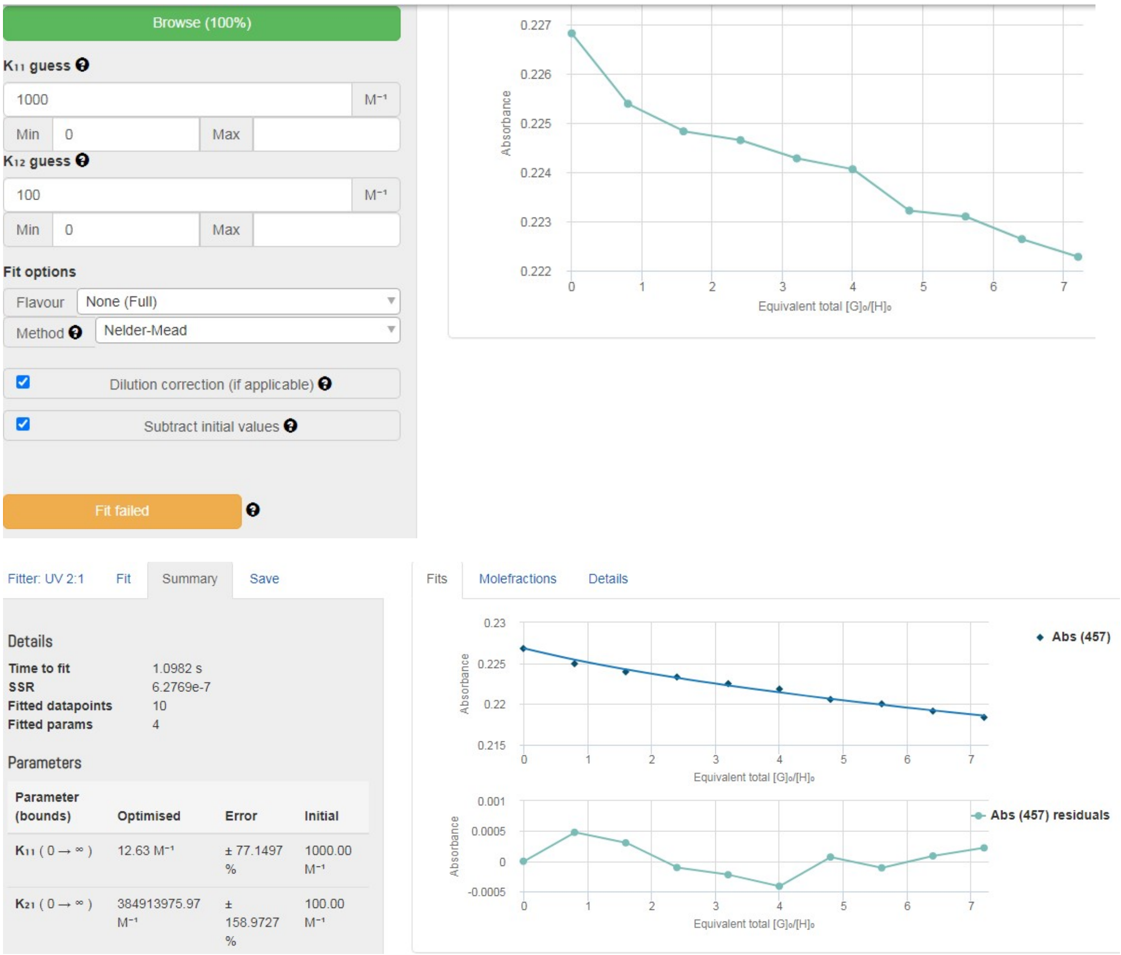

Figure S20. Snapshot capture of Bindfit plots for **C4P7** and TBABr titration, displaying 1:1 stoichiometry with satisfied value of K_{11}, error <2.64% utilizing Nelder-Mead fit.

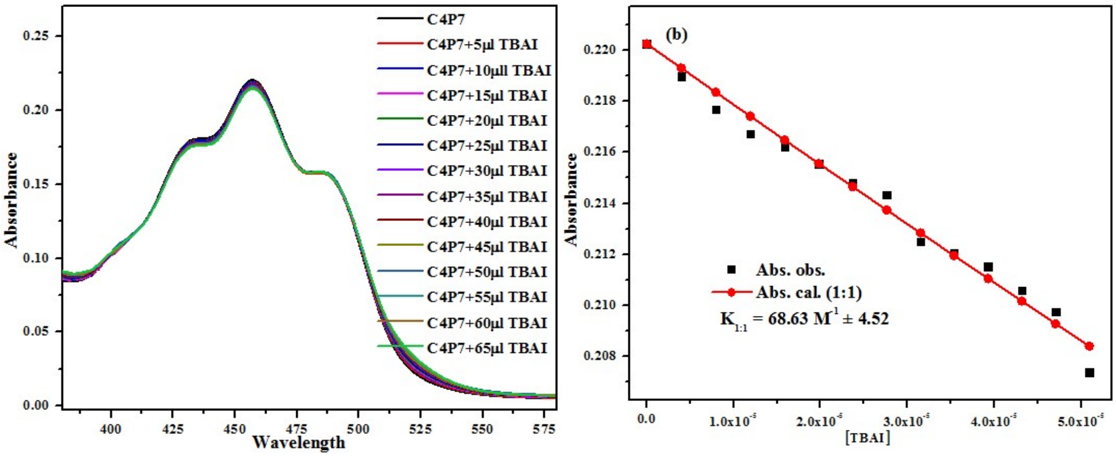

Figure S21. UV-vis titration of receptor **C4P7** with TBAI in CH_3CN and binding isotherm fitting of UV-vis titration data by using Bindfit v0.5 program.

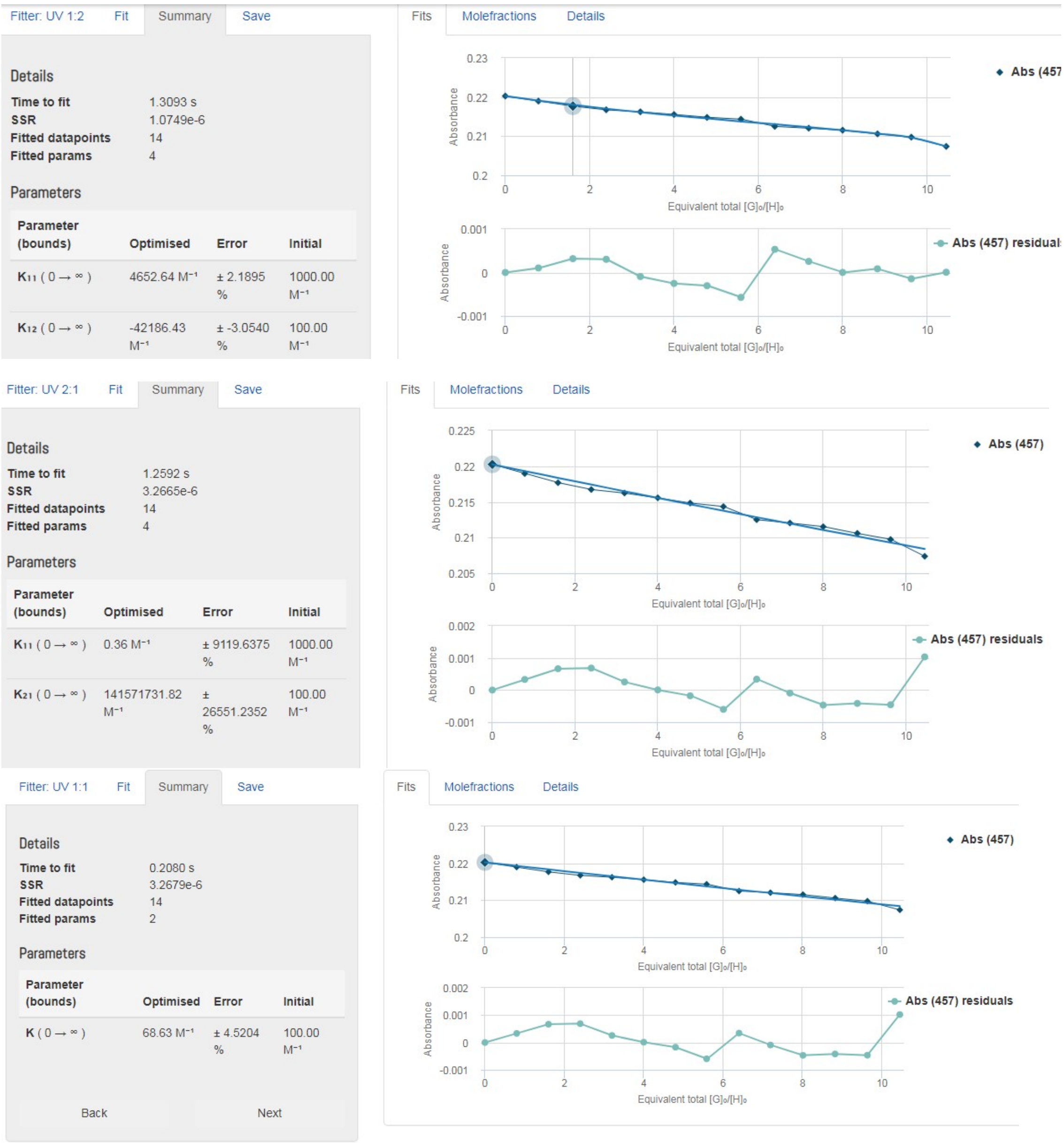

Figure S22. Snapshot capture of Bindfit plots for **C4P7**and TBAI titration, displaying 1:1 stoichiometry with satisfied value of K_{11}, error <4.52% utilizing Nelder-Mead fit.

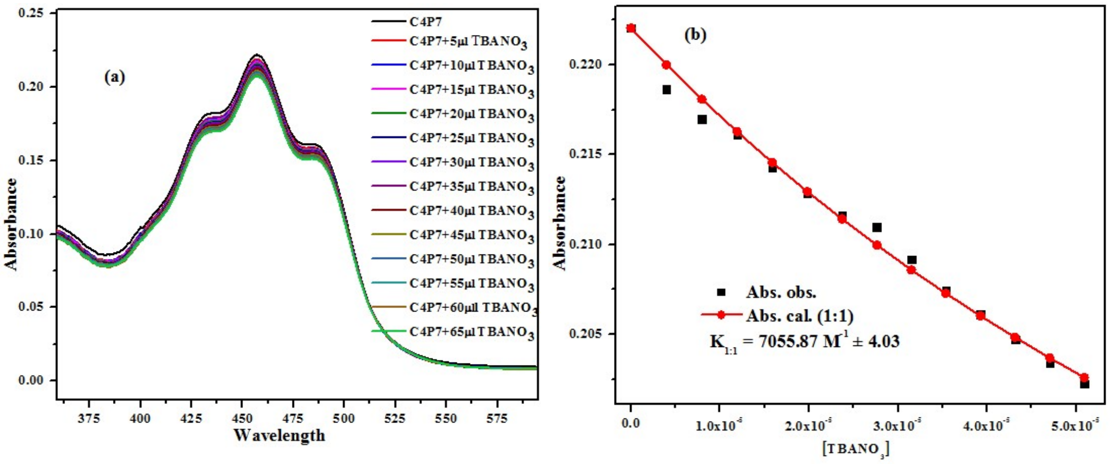

Figure S23. UV-vis titration of receptor **C4P7** with $TBANO_3$ in CH_3CN and binding isotherm fitting of UV-vis titration data by using Bindfit v0.5 program.

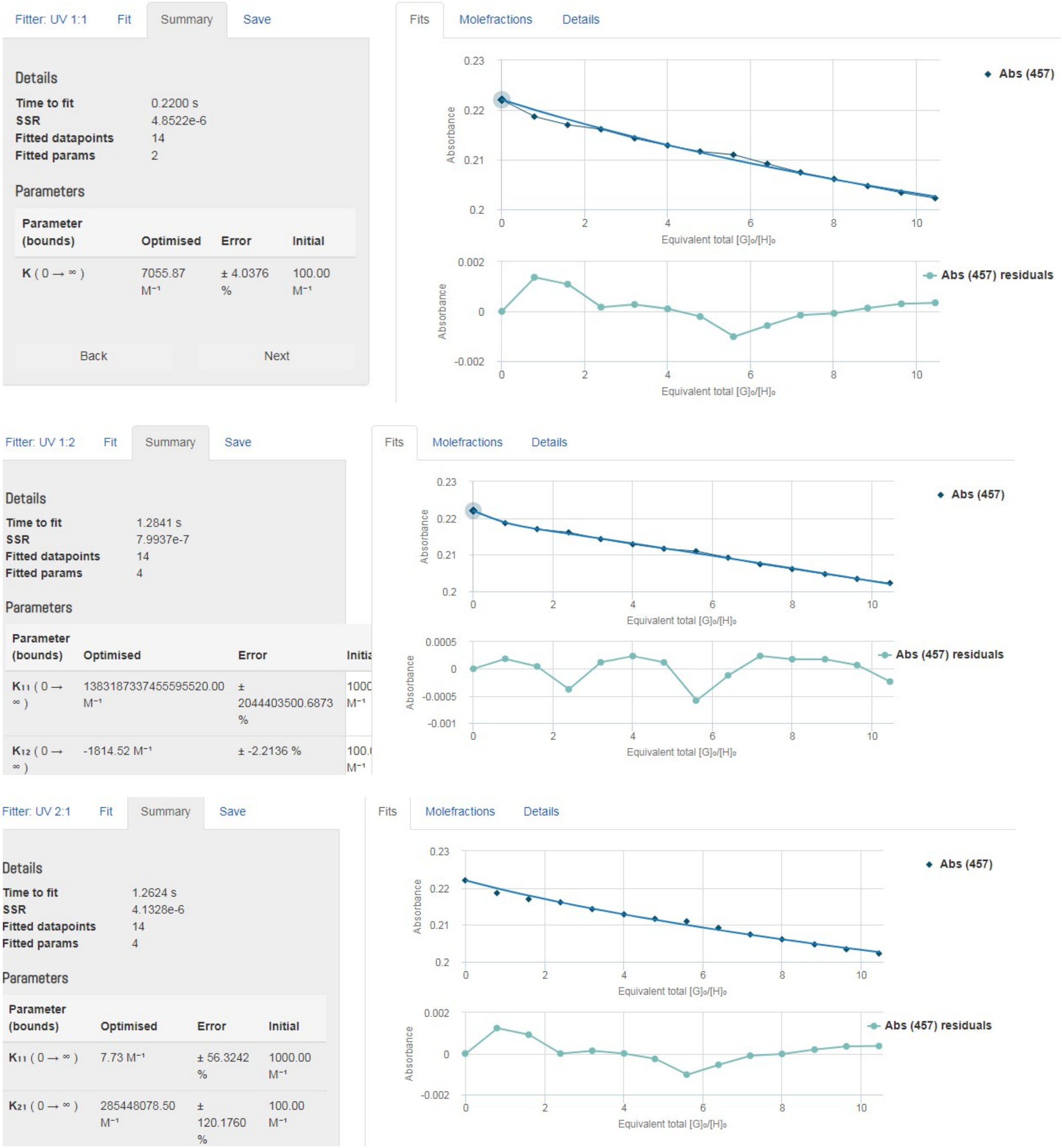

Figure S24. Snapshot capture of Bindfit plots for **C4P7** and $TBANO_3$titration, displaying 1:1 stoichiometry with satisfied value of K_{11}, error <4.03% utilizing Nelder-Mead fit.

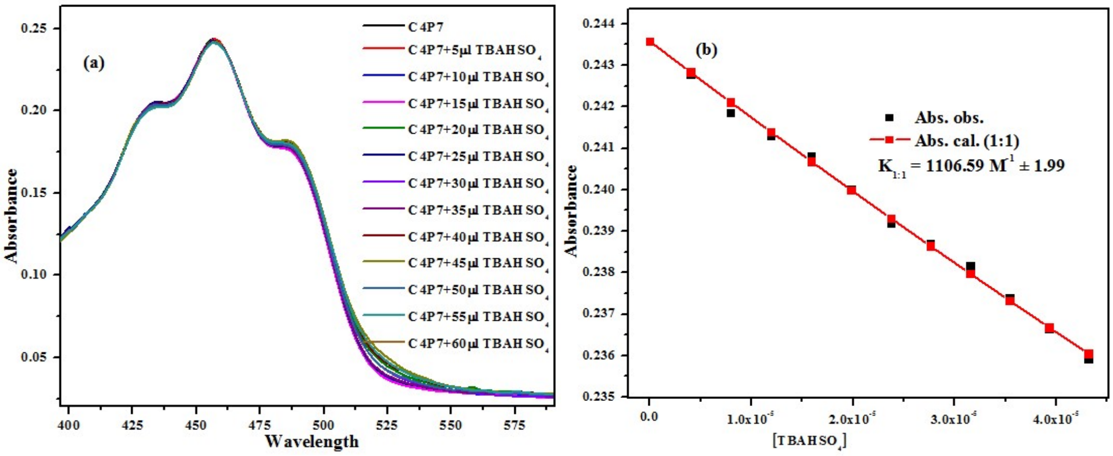

Figure S25. UV-vis titration of receptor **C4P7** with $TBAHSO_4$ in CH_3CN and binding isotherm fitting of UV-vis titration data by using Bindfit v0.5 program.

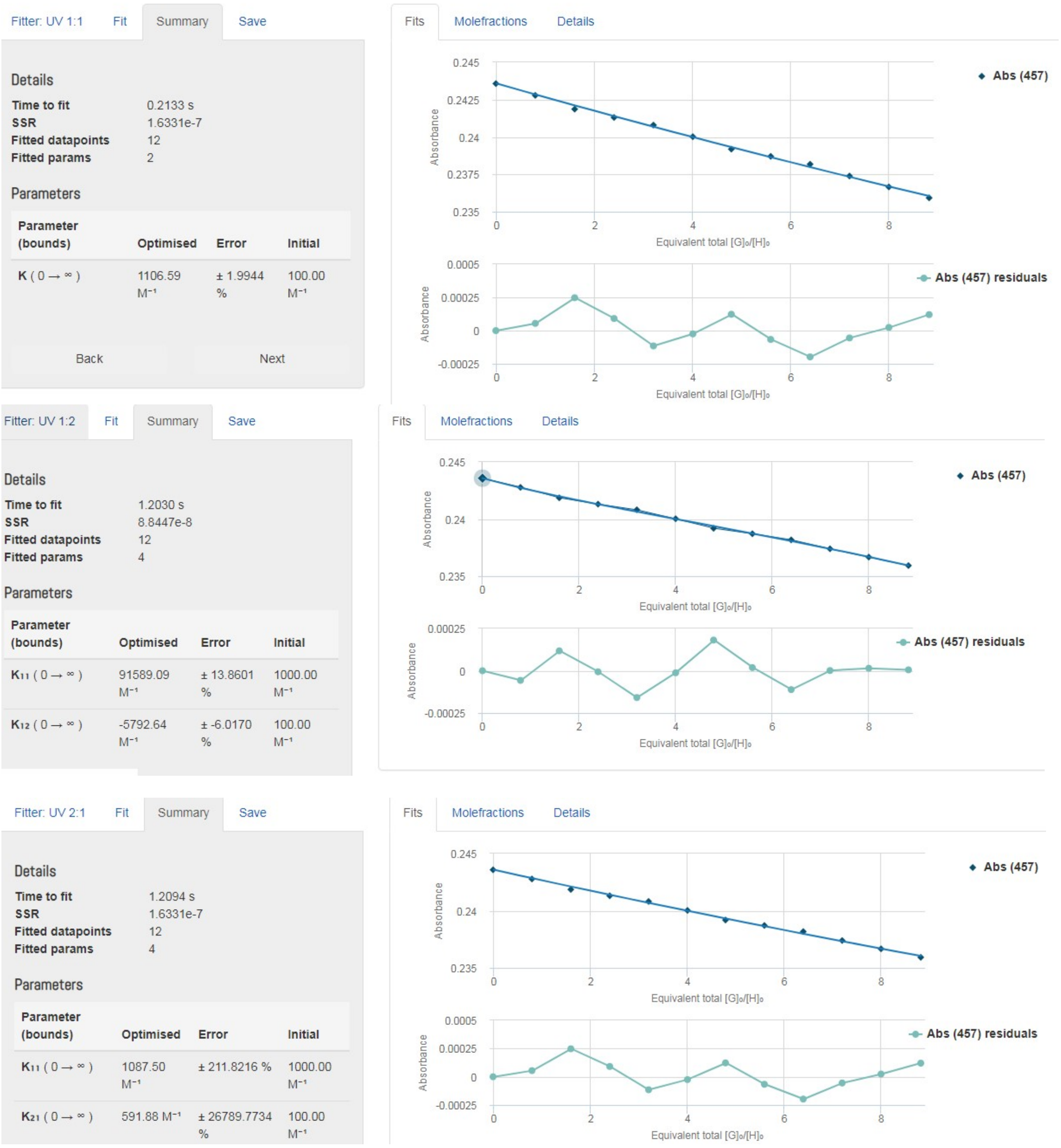

Figure S26. Snapshot capture of Bindfit plots for **C4P7** and $TBAHSO_4$ titration, displaying 1:1 stoichiometry with satisfied value of K_{11}, error <1.99% utilizing Nelder-Mead fit.

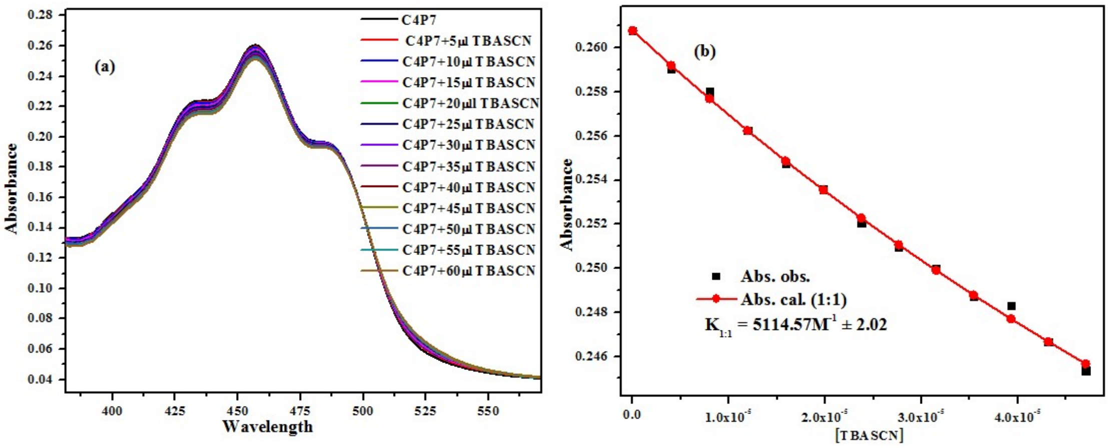

Figure S27. UV-vis titration of receptor **C4P7** with TBASCN in CH_3CN and binding isotherm fitting of UV-vis titration data by using Bindfit v0.5 program.

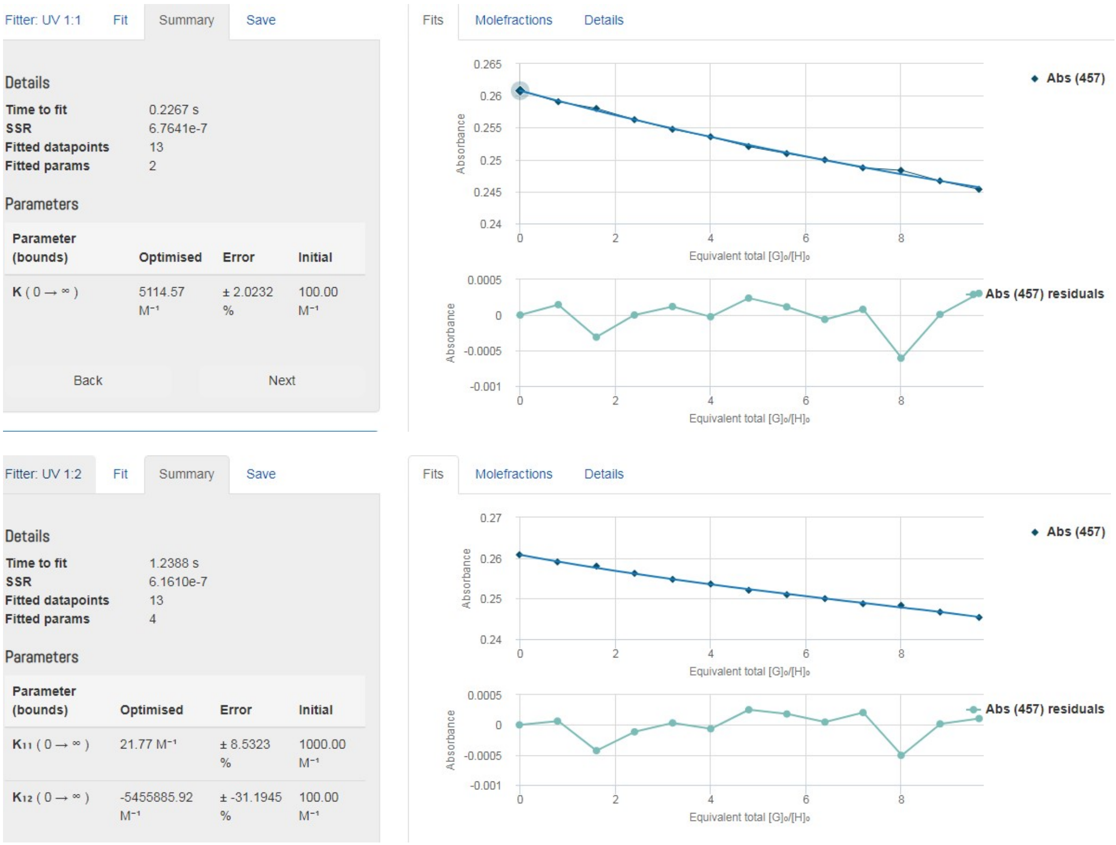

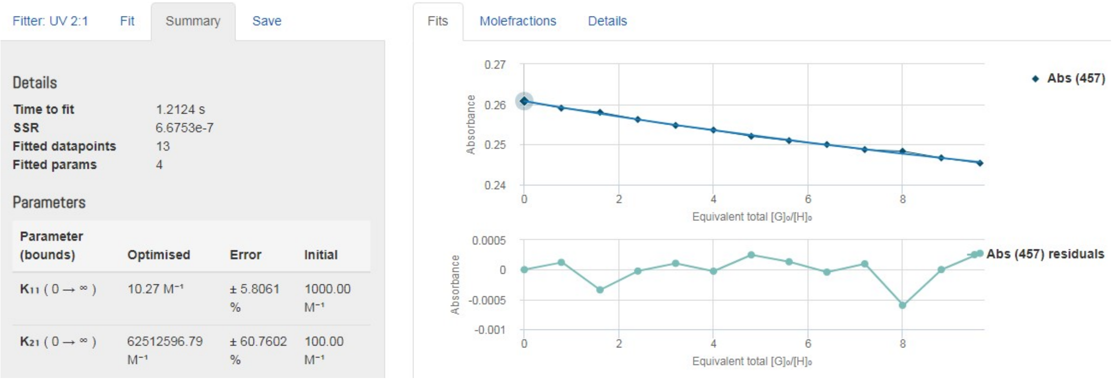

Figure S28. Snapshot capture of Bindfit plots for **C4P7** with TBASCN titration, displaying 1:1 stoichiometry with satisfied value of K_{11}, error <2.02% utilizing Nelder-Mead fit.

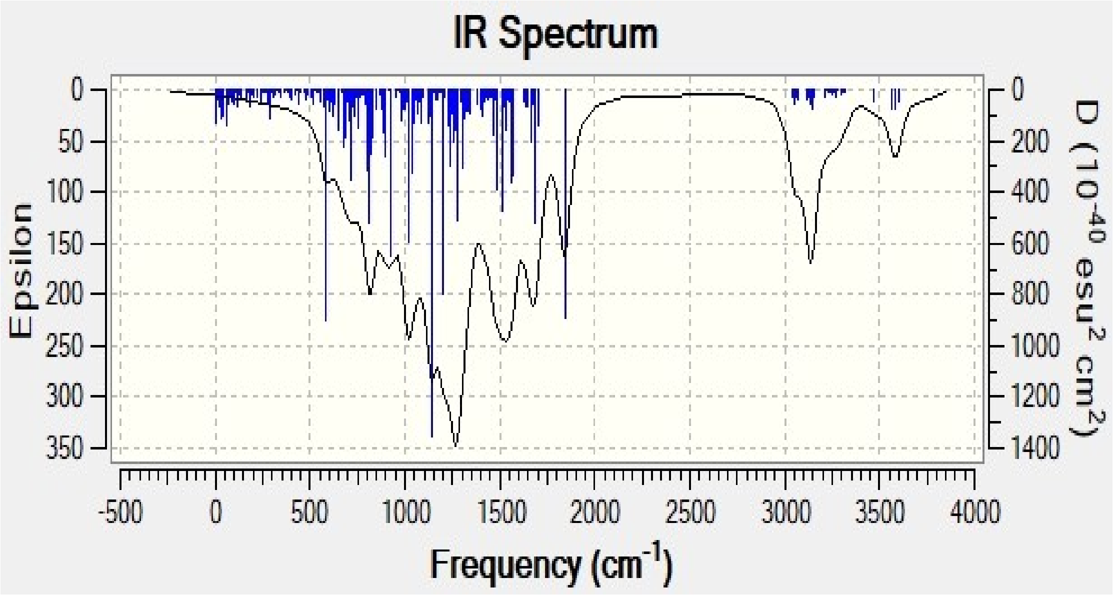

Figure S29. Theoretically predicted IR spectra of **C4P7.**

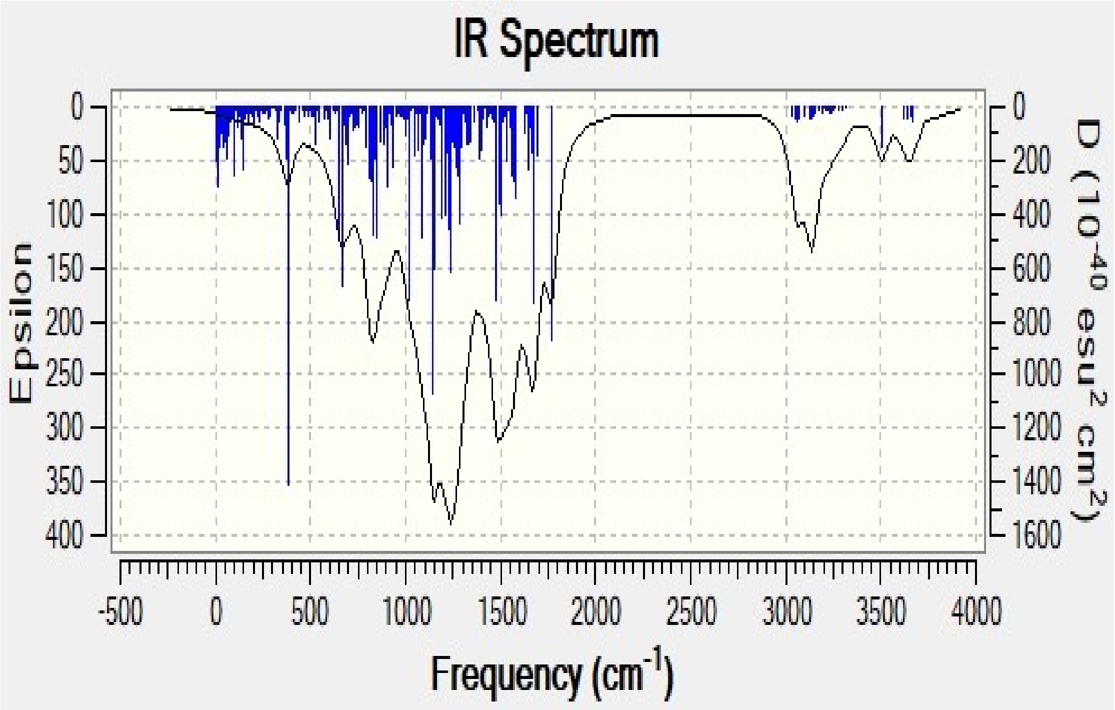

Figure S30. Theoretically predicted IR spectra of **C4P7-Cl.**

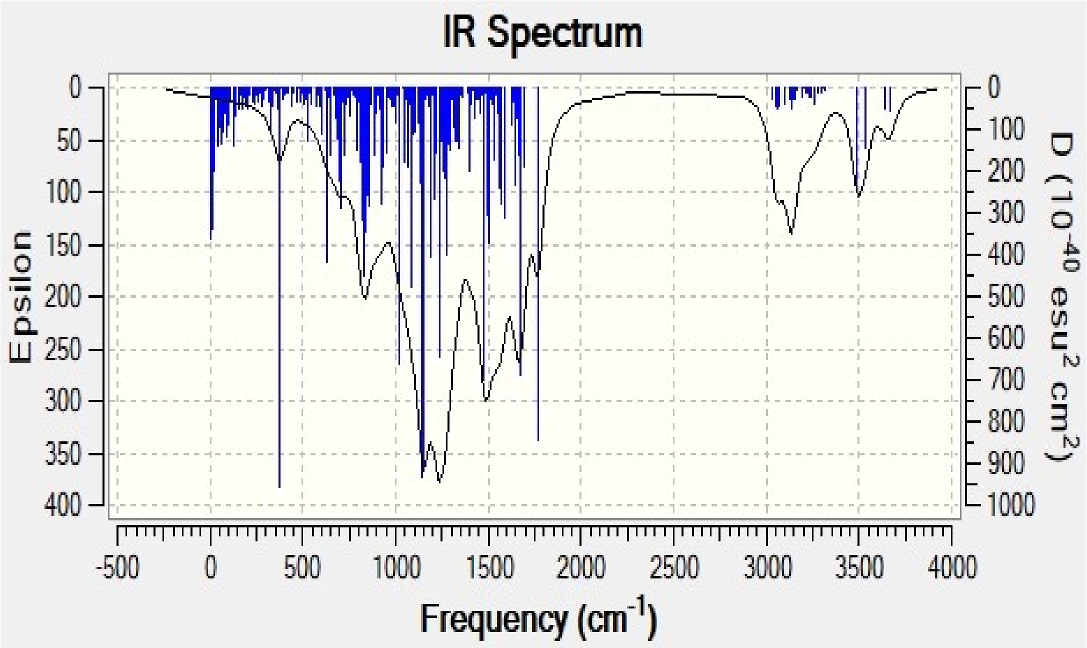

Figure S31. Theoretically predicted IR spectra of **C4P7-**Br.

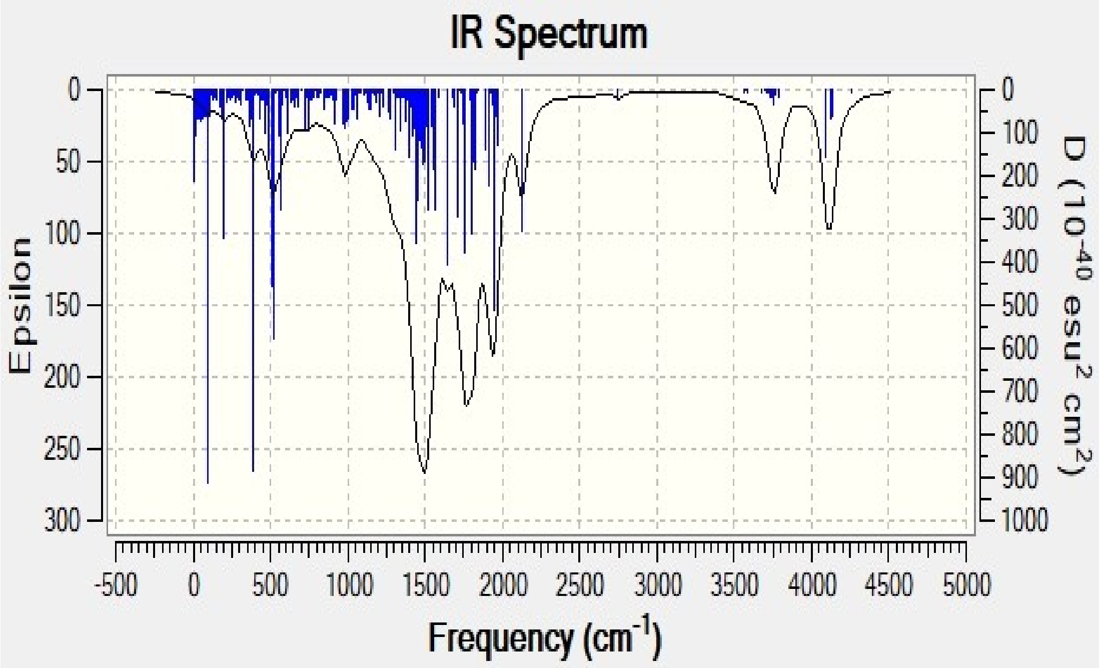

Figure S32. Theoretically predicted IR spectra of **C4P7-**I.

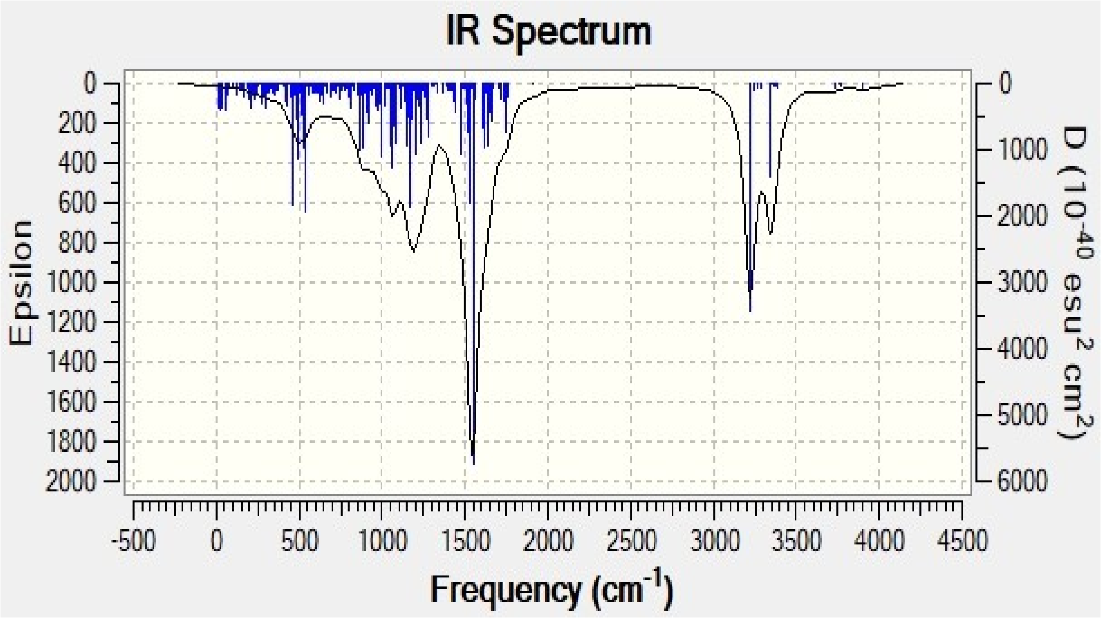

Figure S33. Theoretically predicted IR spectra of **C4P7**-NO_3.

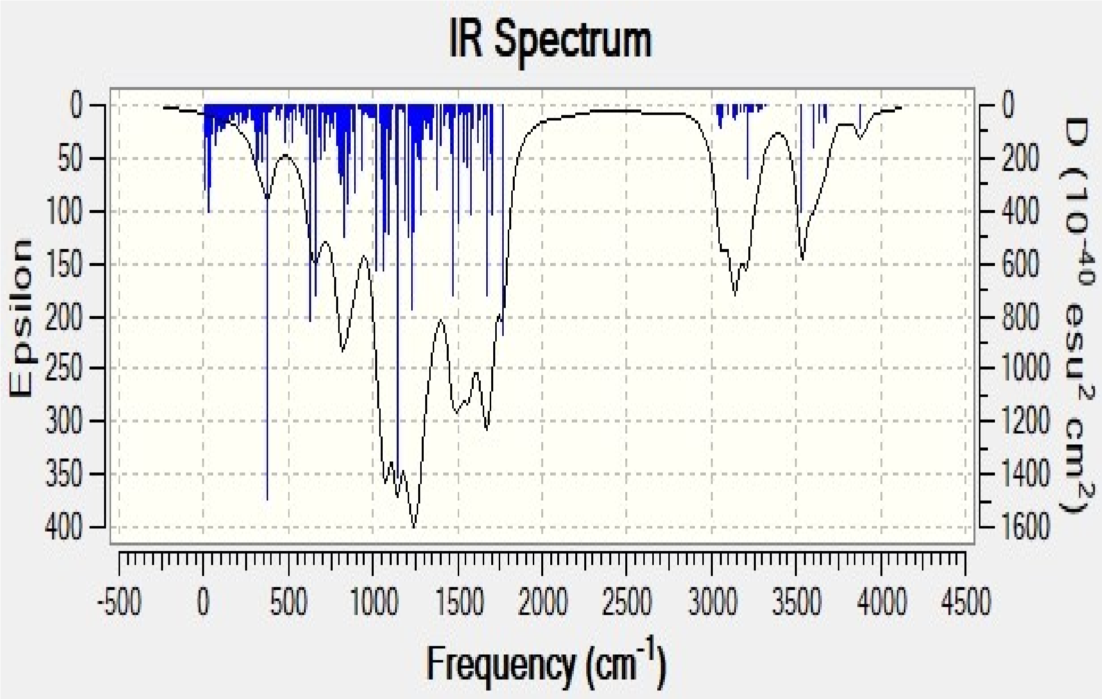

Figure S34. Theoretically predicted IR spectra of **C4P7**-HSO_4.

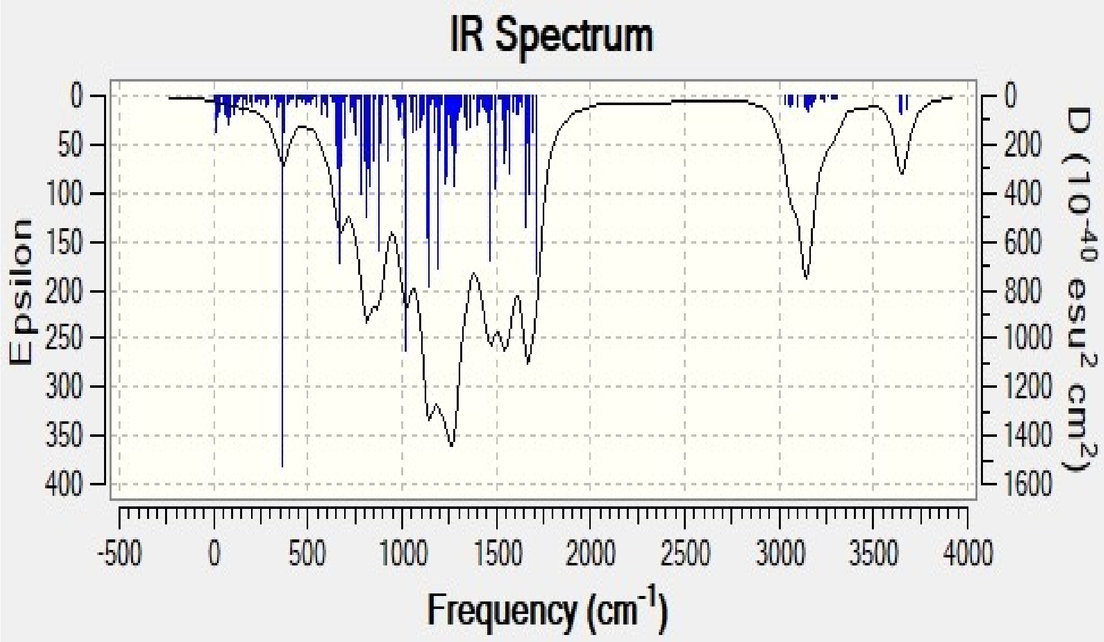

Figure S35. Theoretically predicted IR spectra of **C4P7**-SCN.

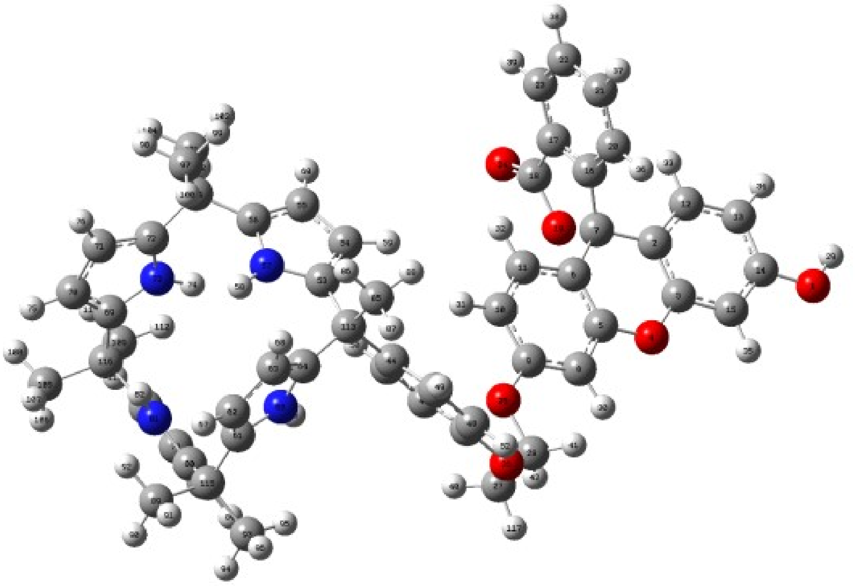

Figure S36. DFT optimized structures of the **C4P7** using 6–31G(d) basis set.

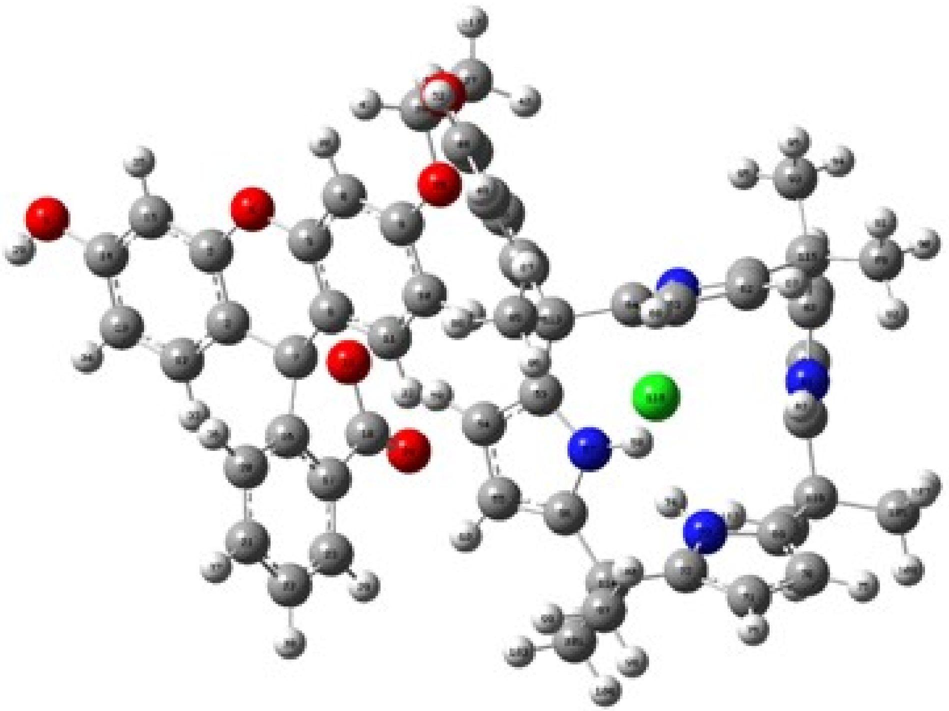

Figure S37. DFT optimized structures of the receptor **C4P7** with TBACl salts using 6–31G(d) basis set. TBA counter cations have been omitted for clarity.

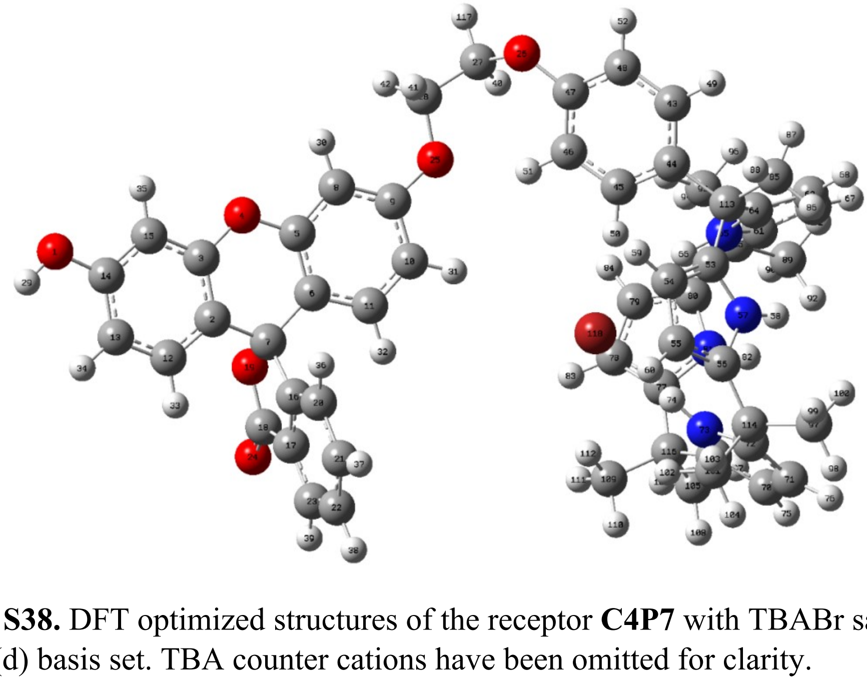

Figure S38. DFT optimized structures of the receptor **C4P7** with TBABr salts using 6–31G(d) basis set. TBA counter cations have been omitted for clarity.

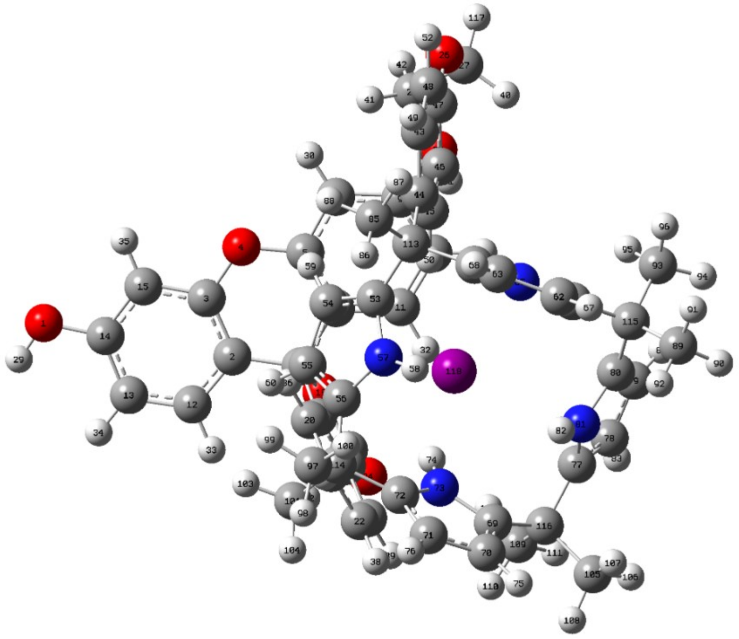

Figure S39. DFT optimized structures of the receptor **C4P7** with TBAI salts using 6–31G(d) basis set. TBA counter cations have been omitted for clarity.

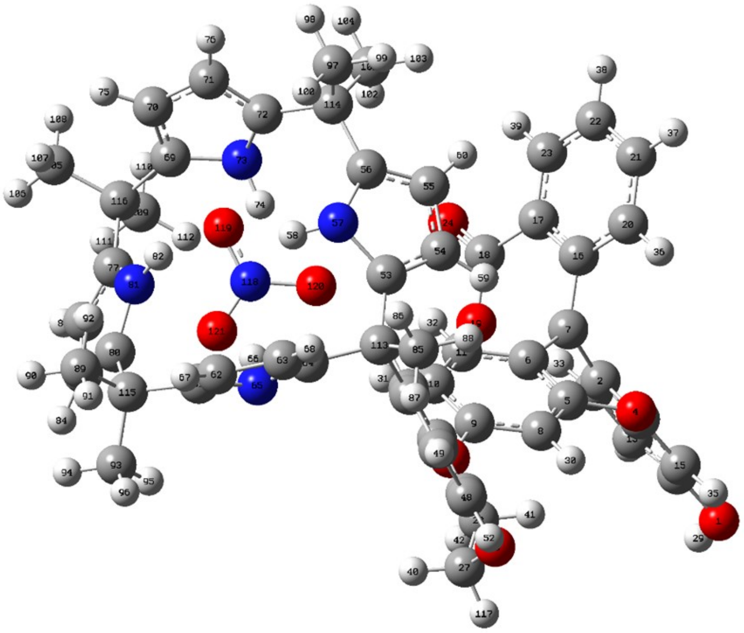

Figure S40. DFT optimized structures of the receptor **C4P7** with TBANO_3salts using 6–31G(d) basis set. TBA counter cations have been omitted for clarity.

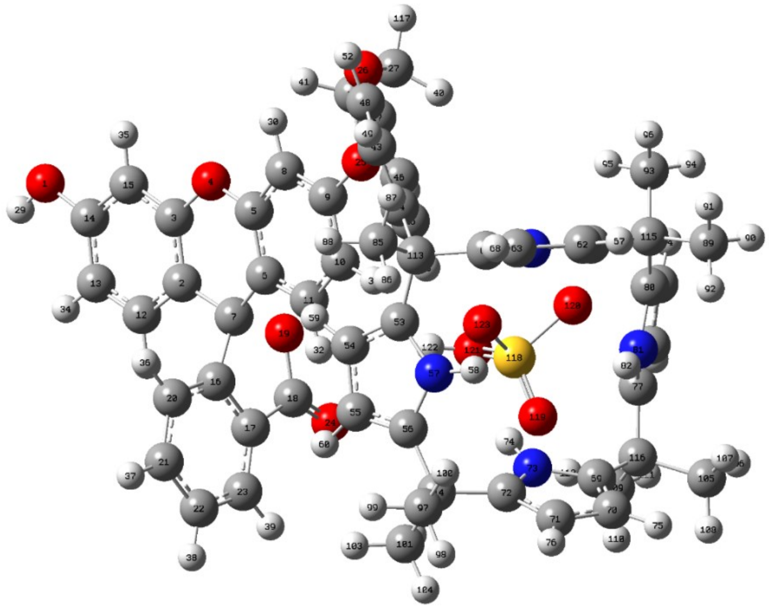

Figure S41. DFT optimized structures of the receptor **C4P7** with TBAHSO$_4$ salts using 6–31G(d) basis set. TBA counter cations have been omitted for clarity.

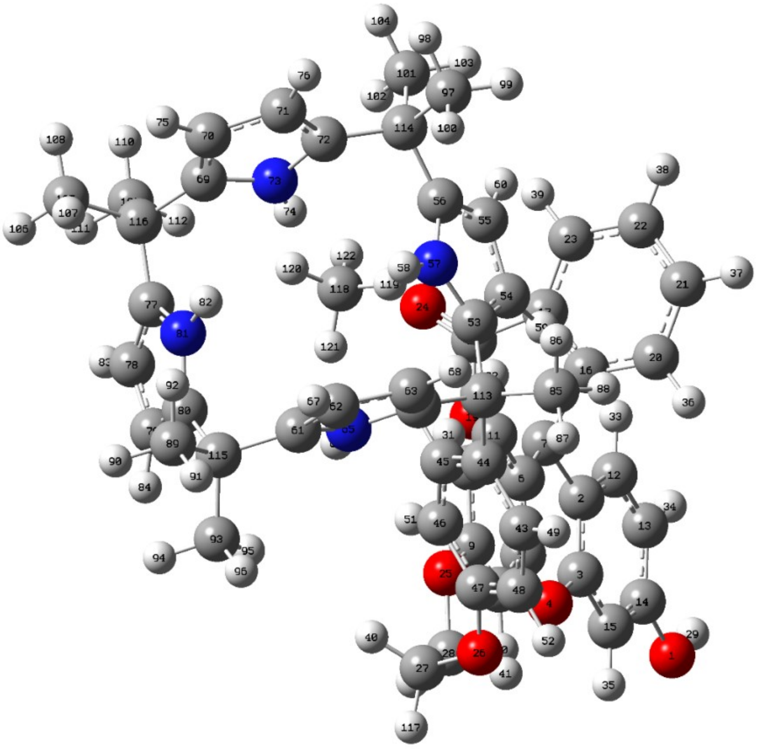

Figure S42. DFT optimized structures of the receptor **C4P7** with TBASCN salts using 6–31G(d) basis set. TBA counter cations have been omitted for clarity.

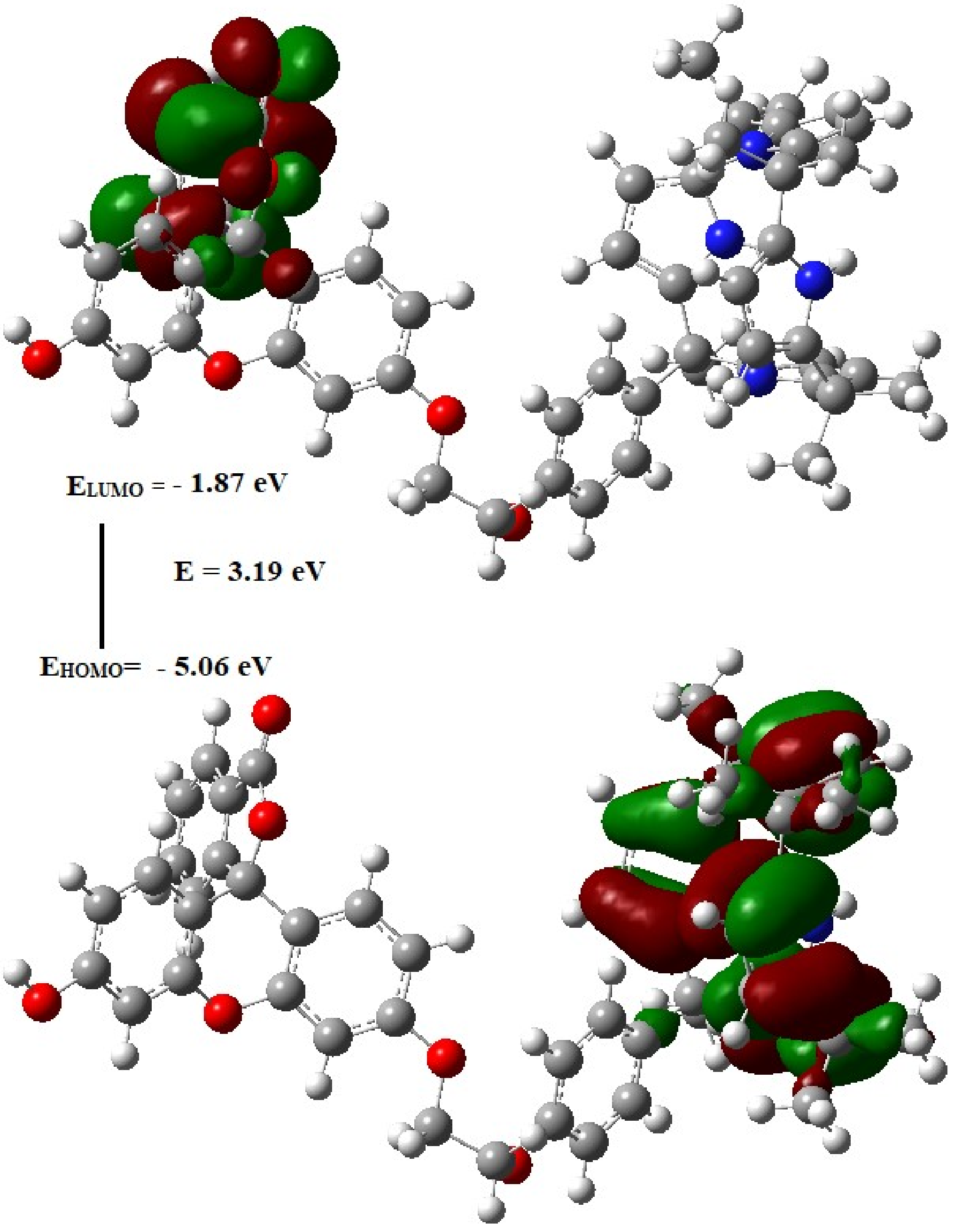

Figure S43. Frontier molecular orbital distribution of **C4P7**, calculated with the help of TD-DFT using 6–31G(d) basis set.

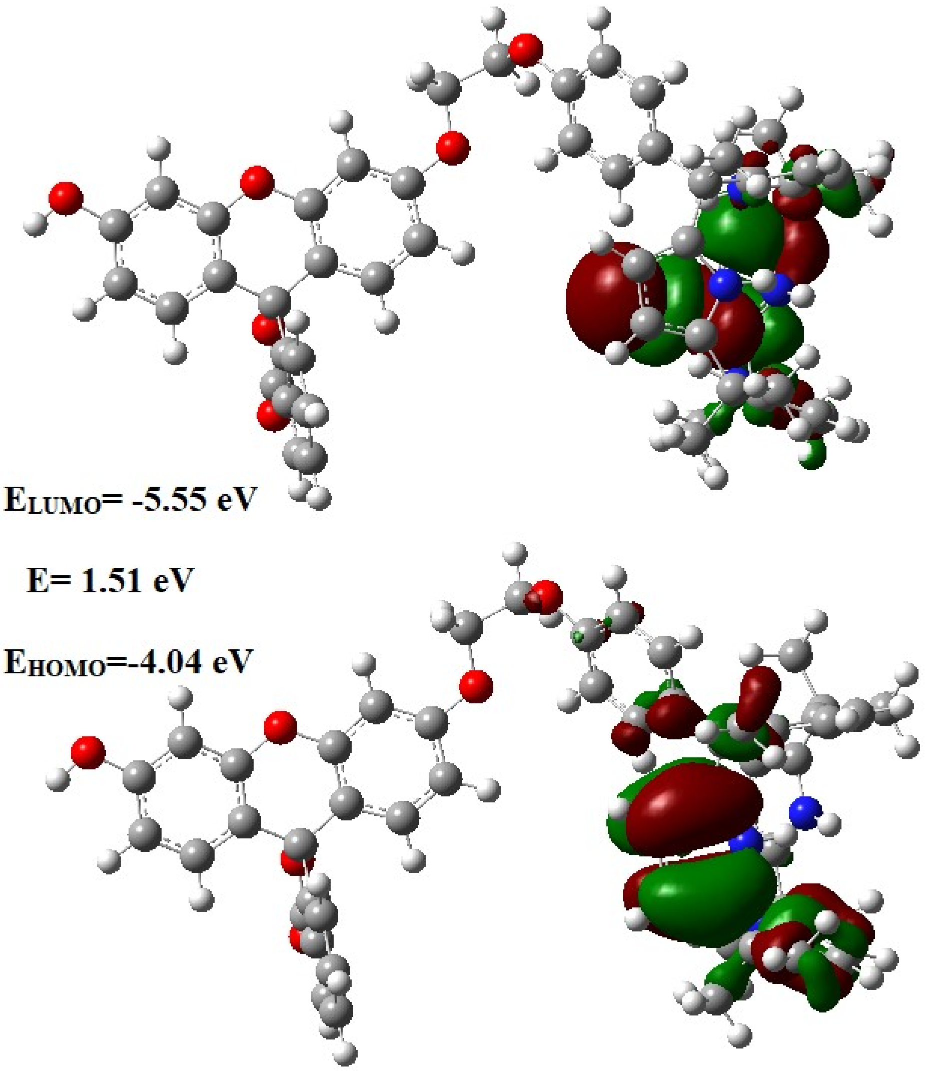

Figure S44. Frontier molecular orbital distribution of **C4P7-**TBACl; calculated with the help of TD-DFT using 6–31G(d) basis set.

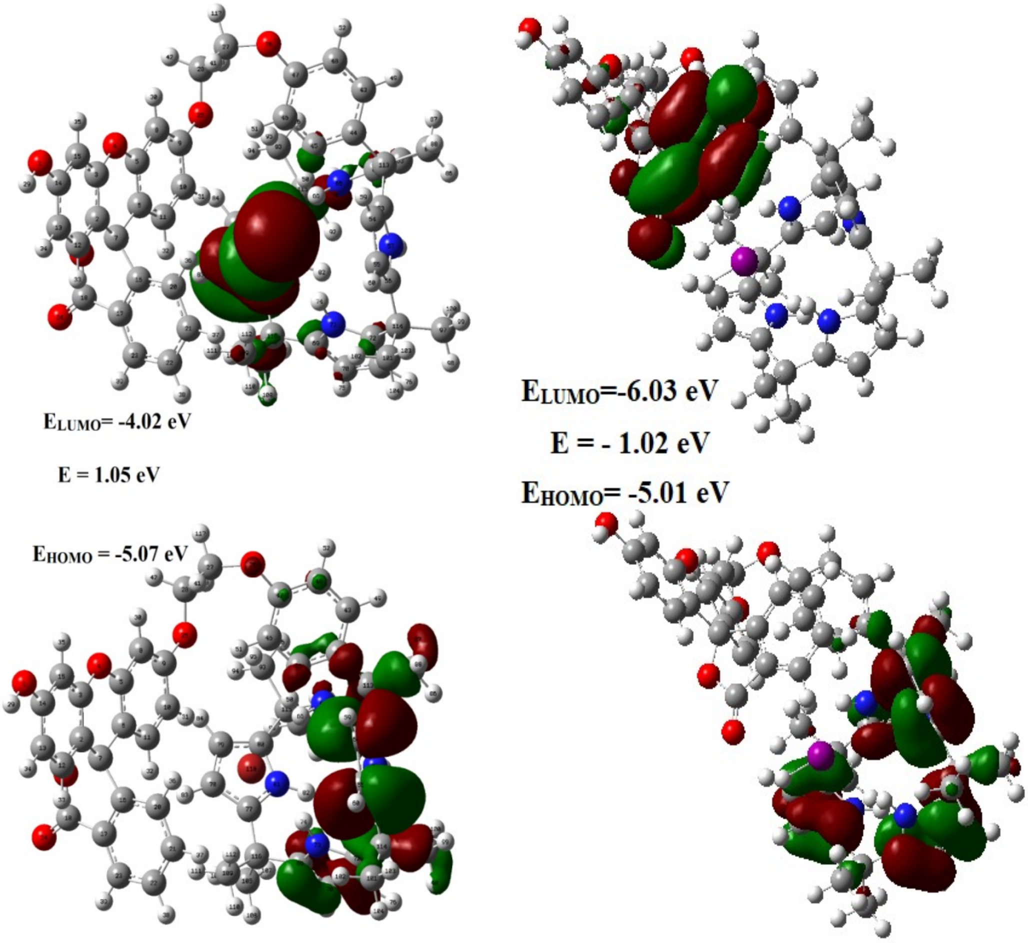

Figure S45. Frontier molecular orbital distribution of **C4P7-**TBABr and **C4P7-**TBAI; calculated with the help of TD-DFT using 6–31G(d) basis set.

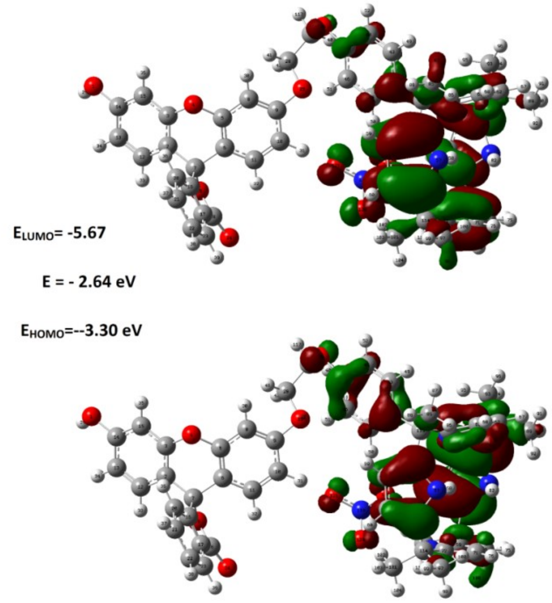

Figure S46. Frontier molecular orbital distribution of **C4P7-**TBANO_3 calculated with the help of TD-DFT using 6–31G(d) basis set.

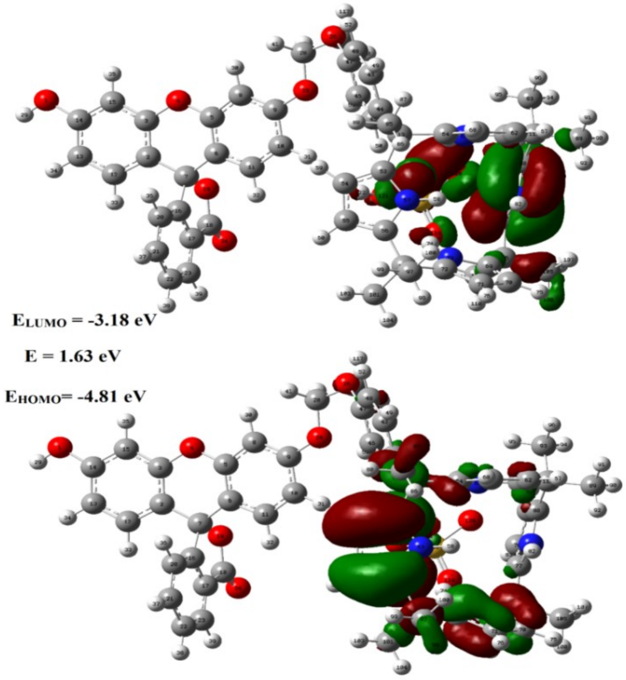

Figure S47. Frontier molecular orbital distribution of **C4P7-**$TBAHSO_4$ calculated with the help of TD-DFT using 6–31G(d) basis set.

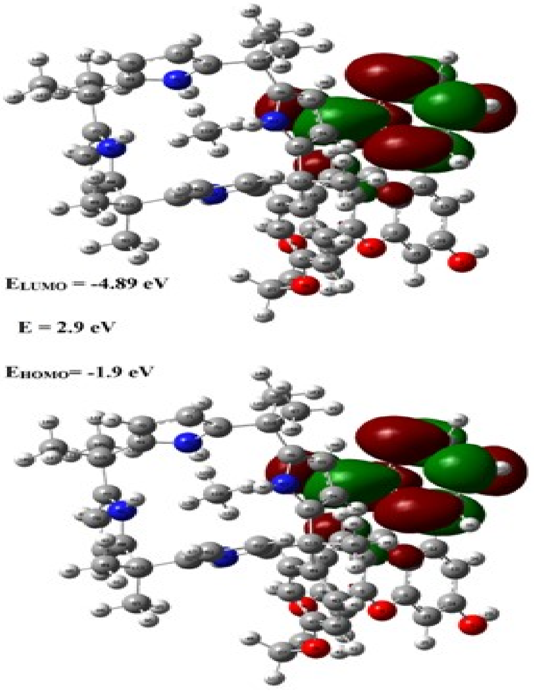

Figure S48. Frontier molecular orbital distribution of **C4P7-**TBASCN calculated with the help of TD-DFT using 6–31G(d) basis set.

References

1. P. Thordarson, *Chem. Soc. Rev.*, 2011, **40**, 1305–1323.

2. P. A. Gale and J. W. Steed, Eds., *Supramolecular Chemistry*, John Wiley & Sons, Ltd, Chichester, UK, 2012.

1. I. A. Rather and R. Ali, *Green Chem.*, 2021, **23**, 5849-5855.

2. S. A. Wagay, R. Ali, Facile synthesis and anion binding studies of fluorescein/benzo12-crown-4 ether based bis-dipyrromethane (DPM) receptors. RSC Adv., 2023, **13**, 30420-30428.
